# Bioinspired chitosan based functionalization of biomedical implant surfaces for enhanced hemocompatibility, antioxidation and anticoagulation potential: an *in silico* and *in vitro* study†

**DOI:** 10.1039/d4ra00796d

**Published:** 2024-07-01

**Authors:** Sadia Hassan, Namra Bilal, Tooba Javaid Khan, Murtaza Najabat Ali, Bakhtawar Ghafoor, Khawaja Usman Saif

**Affiliations:** a School of Mechanical and Manufacturing Engineering (SMME), National University of Sciences and Technology Islamabad Pakistan shassan.phd20smme@student.nust.edu.pk; b Nencki Institute of Experimental Biology Poland; c Isra University Islamabad Pakistan

## Abstract

Endowing implanted biomaterials with better hemocompatibility, anticoagulation, antioxidant and antiplatelet adhesion is necessary because of their potential to trigger activation of multiple reactive mechanisms including coagulation cascade and potentially causing serious adverse clinical events like late thrombosis. Active ingredients from natural sources including *Foeniculum vulgare*, *Angelica sinensis*, and *Cinnamomum verum* have the ability to inhibit the coagulation cascade and thrombus formation around biomedical implants. These properties are of interest for the development of a novel drug for biomedical implants to potentially solve the current blood clotting and coagulation problems which lead to stent thrombosis. The objective of this study was to incorporate different anticoagulants from natural sources into a degradable matrix of chitosan with varying concentrations ranging from 5% to 15% and a composite containing all three drugs. The presence of anticoagulant constituents was identified using GC-MS. Subsequently, all the compositions were characterized principally by using Fourier transform infrared spectroscopy and scanning electron microscopy while the drug release profile was determined using UV-spectrometry for a 30 days immersion period. The results indicated an initial burst release which was subsequently followed by the sustained release pattern. Compared to heparin loaded chitosan, DPPH and hemolysis tests revealed better blood compatibility of natural drug loaded films. Moreover, the anticoagulation activity of natural drugs was equivalent to the heparin loaded film; however, through docking, the mechanism of inhibition of the coagulation cascade of the novel drug was found to be through blocking the extrinsic pathway. The study suggested that the proposed drug composite expresses an optimum composition which may be a practicable and appropriate candidate for biomedical implant coatings.

## Abstract 2

Mitigating thrombosis and adverse reactions in biomedical implants necessitates exploration of natural biomaterials with enhanced hemocompatibility and anticoagulation potential including natural drug-loaded chitosan based matrixes.

## Graphical abstract

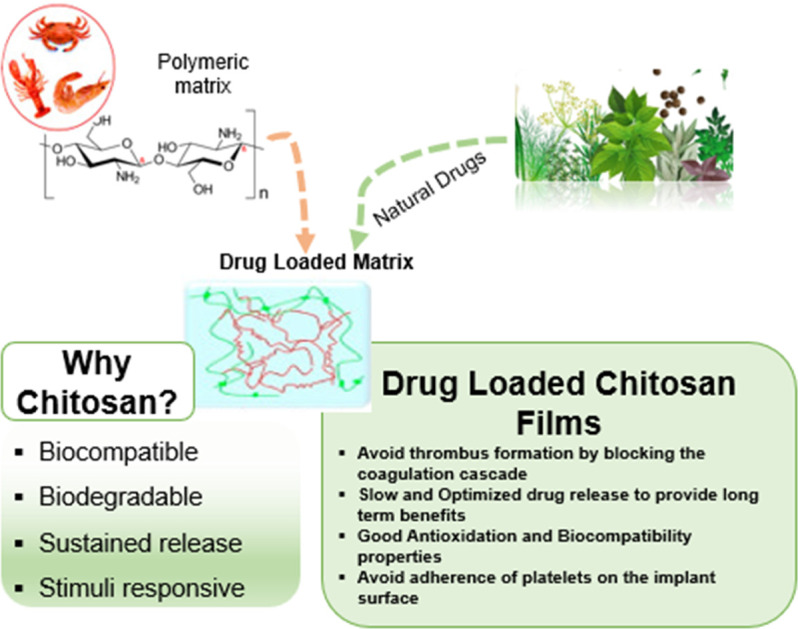

## Introduction

Coupled with daily population habits and a longer life expectancy of humans, the rate of malfunctioning of human body parts has increased in recent years which ultimately increased the use of biomedical implants to replace the damaged part of the body to maintain and prolong a healthy lifestyle. The statistics have demonstrated that 7.9 million Americans in 2020 received joint implants and around one million received metallic knee and hip implants to keep a normal lifestyle.^1^ In addition, there was a high number of pacemaker implantations, *i.e.*, 370 000 (ref. 2) and 600 000 cardiac stents.^3^ The prevalence of chronic diseases and increased number of accidental injuries have made biomedical implants a need of hour and research is being conducted to improve the medical outcomes and improved efficiency of the treatment.

There has been extensive research for decades to improve the surfaces and materials of medical implants to decrease the rate of rejections which were caused by the host immune response, thrombo-inflammatory cascades, infections, mechanical and biocompatibility issues and allergic reactions.^4^ Therefore, the materials of implants, their framework and surfaces are modified in such a way that these do not protect their structural and compositional integrity against well-orchestrated thrombo-inflammatory cascades but also inhibit the activation of the coagulation cascade, complement system, cellular inflammatory mechanisms, and platelets.^5^ There are many ways to enhance the compatibility of medical devices with the body, *i.e.*, structural, or chemical modifications of implant surfaces, and these modifications have already been proposed in literature. For example, Asadi *et al.* used a combination of silk fibroin and cellulose nanocrystals to increase the corrosion resistance orthopedic implants.^6^ In our previous study, we used graphite-based hard materials to enhance the anticoagulation and biocompatibility of the medical implants.^7^ There are also bioactive strategies which use pharmacologic active ingredients for the inhibition of the coagulation and immune response by local drug delivery or permanent immobilization of an active agent on the surface.^8,9^

Thrombosis is one of the major issues which are responsible for the adverse effects of implants and their failure. The platelet activation and coagulation around the implant can block the blood flow and isolate the implant from the surrounding environment rendering it unable to perform its functions. To reduce the chances of thrombus formation, different anticoagulation based materials are used *i.e.*, heparin which has been found to be most efficient in reducing the thrombus formation by over 70%.^10^ Despite of its efficiency, it has been associated with heparin-induced thrombocytopenia which can increase the risk of clots forming around the coated surface.^11^ Many other anticoagulants including warfarin,^12^ aspirin,^13^ and acenocoumarol^14^ have been reported to be used for biomedical implants applications to reduce the rate of thrombosis. Nevertheless, these molecules have high potency, short-term effects and chances of severe bleeding which makes them an unfavorable choice.

In recent years, scientists have provided alternatives to heparin by introducing low molecular weight heparin,^15,16^ antithrombin heparin^17,18^ and natural anticoagulants.^19^ There has been a keen interest in natural anticoagulants because of the lower risks of adverse events. Among natural anticoagulants includes *A. sativum*,^20^*C. longa*,^21^*C. cassia*,^22^*A. sinensis*,^23^*P. ginseng*^24^ and *O. tenuiflorium*^25^ which are well reported for their effectiveness and feasibility in biomedical applications. In our latest study, we reported the anticoagulation effects of individual and combined effects of *C. cassia*, *O. tenuiflorium* and *P. ginseng* for biomedical applications^9^ and in this study, we focused on *C. verum*, *A. sinensis* and *F. vulgare* based drugs and tried to explore their suitability for biomedical implants applications.

Plant based drugs have always been a point of focus because of their mild efficacy, effectiveness and compatibility with human body. *C. verum* (*Cinnamomum verum*) is an Asian spice and commonly found in Indian, Chinese and Sri Lankan regions. It has been famous for its antioxidant, anti-inflammatory, and antimicrobial properties. There are different studies in literature which demonstrated the benefits of this spice in different diseases including diabetes,^26^ infections,^27^ cancer,^28^ and wound healing.^29^ These properties come from its components including cinnamaldehyde, cinnamic acid, coumarins, diterpenoids, and polyphenols.^30,31^ Like cinnamon, *A. sinensis* has many medicinal properties due to ferulic acid and *Z*-ligustilide being the major actives in the extract. It has anti-inflammatory and anti-platelet aggregation abilities. It has been famously used for the treatment of female irregular menstruation and amenorrhea.^32^ Lastly, *Foeniculum vulgare* (commonly known as fennel) is a widely used herb and spice. The Fennel seeds are considered an important source of *trans*-anethole, fenchone, estragole and other ingredients which are responsible for providing protection from infections, digestive problems and reduce inflammation.^33^ There are many studies on literature which showed its therapeutic effects against cardiovascular ailments and cancer.^34,35^

The selection of a suitable drug carrier is a critical step as it works synergistically with the drug to ensure effectiveness of functions of biomedical implants. Looking at commercial coronary implants, initial stents (cypher and taxus) had non-degradable polymeric drug carriers including polyethylene vinyl acetate^36^ and poly(styrene-*block*-isobutylene-*block*-styrene),^37^ respectively. After recognizing the limitations of permanent polymers in first generation of stents, researchers directed their focus towards biodegradable alternatives. Amomg different polymer poly(lactic acid) (PLA) and its derivatives, particularly poly(lactic-*co*-glycolic acid) (PLGA), poly(l-lactic acid) (PLLA), and poly(d,l-lactic acid) (PDLLA), grabbed the attention due to their biocompatibility, flexibility and degradation properties.^36^ These polymers are used in contemporary drug-eluting stents (DES) like orsiro and synergy, which currently dominate the market. A key challenge associated with stents which has PLA is a drug carrier is the discrepancy between degradation time of drug carrier (18–24 months) and the desired drug release window (4–12 months).^38^ Now, extensive research efforts are aimed at using those polymers whose degradation profiles closely align with optimal drug release kinetics *i.e.*, poly(glycolic acid),^39^ chitosan^40^ and polyvinyl alcohol.^41^ Chitosan is a commonly used in biomedical applications due to hydrophilicity, biocompatibility, biodegradability, and bioactivity. Use of chitosan for coronary stents have already been reported in various studies because it has heparin-like polysaccharide structure. In literature, Qiu *et al.*^42^ used chitosan to create the structure of bioresorbable coronary stents. In their study, chitosan was successfully used to develop bioresorbable coronary stents using 3D printing and these stents exhibited a promising degradation profile, with only 10% degradation observed within the first 30 days. This finding highlighted the potential use of chitosan for controlled degradation in biomedical devices. In another study, Meng *et al.*^43^ reported the use of chitosan for coronary stents by making a composite of chitosan with a strong anticoagulant (heparin). This study was aimed to develop a similar composite material utilizing natural anticoagulants instead of synthetic ones. Yang *et al.*^44^ also used Chitosan a drug carrier for coronary stents by combining it with anti-CD34 antibody and sirolimus. The evidence presents in the literature regarding the application of chitosan as a drug carrier for various biomedical applications established its potential for our study. Consequently, chitosan was selected as a polymeric drug carrier for novel anticoagulants in this investigation.

The coating of antiplatelet or anticoagulant drugs on medical implants have gained interest as these drugs are able to either block the coagulation cascade or decrease the platelet adhesion capability and ultimately decreasing the rate of thrombus formation.^45–47^ In this study, we are exploring the anticoagulation behavior of, *A. sinensis*, *F. vulgare* and *C. verum* for the application of biomedical implants coatings to avoid the issue of thrombosis at the implant site. There have been numerous studies in which their anticoagulation and anti-oxidative behavior potential was investigated;^48–51^ nevertheless, there is a lack of literature on the applications for biomedical implants as coating material. In this study, drug loaded polymeric matrixes were fabricated and optimized to impart the anticoagulation properties in medical implants. The novelty of the project lies in the drug-polymer combinations and their applications in medical implants. Lastly, these novel polymeric compositions may be served as an alternative to synthetic drugs and reduce the issues associated with them. Furthermore, this will also give us an opportunity to explore the behavior and interaction of drugs which is still an untapped field.

## Methodology

### Materials

The bark of *Cinnamon verum* (Cinnamon) was obtained from Spice Mountain (London) and *Angelica sinensis* (commonly known as female ginseng or dong quai) was provided by Drotrong Chinese Herb Biotech Co., Ltd (Chengdu, China). The dried seeds of *Foeniculum vulgare* were provided by a local vendor. Chitosan powder (M.W. 190 kDa), phosphate buffer saline (PBS) tablets, DPPH (2,2-diphenyl-1-picryl-hydrazyl-hydrate), CaCl_2_, plasma thrombin reagent and actin-activated cephthaloplastin was obtained from Sigma-Aldric. Acetic acid was provided by Fisher Scientific (Loughborough, LE, UK) and used as it was received. For blood-based analysis, human blood was provided by Holy Family Hospital, Pakistan.

### Drug extraction

The drugs were extracted by following different methods as described in literature. The details of extraction method for all the drugs are given below.

### *Foeniculum vulgare* (*F. vulgare)*

The *F. vulgare* seeds were ground and sieved through a mesh (80 mesh) to obtain a fine powder. Afterwards, 20 g of powder was mixed with 20 mL ethanol (100%) at room temperature in an orbital shaker. After 8 hours, the mixture was filtered using Whatman No. 1 filter paper and extract was obtained in liquid form. The process was repeated twice to extract maximum seed contents. The liquid extract was placed in a rotary evaporator at 45 °C to evaporate excess solvent. The remaining solid content was dissolved in PBS and stored at 4 °C for further use.^52^

### *Angelica sinensis* (*A. sinensis)*

The root of *A. sinensis* was ground into a fine powder and sieved using a mesh (80 mesh). The powder was placed in a boiling ethanol solution for 1.5 hours. After filtration, the solvent evaporated and the remaining solid was obtained. It was dissolved in PBS solution and stored at 4 °C for further use.^53^

### *Cinnamomum verum* (*C. verum*)

To obtain the extract of *C. verum*, instructions of ref. 54 were followed. To explain briefly, the cinnamon was grinded into a powder which was soaked in 50% ethanol. Twenty grams of herbal powder was dissolved in 100 mL of ethanol in a 250 mL erlenmeyer flask for 48 hours at 25 °C with frequent shaking. To avoid contamination, the flasks were sealed with a cotton plug and aluminum foil. After obtaining the ethanolic mixture, it was centrifuged at 3500 RPM for 20 min. At the end the solution was filtered through Whatmann filter paper No.1 (Azoro, 2000) and filtrate were obtained which was further concentrated under reduced pressure in a rotary vacuum evaporator to collect a semi-solid substance. The semi-solid was further dried in a crucible under a controlled temperature (45 °C) to obtain a drug powder.

### Preparation of drug loaded films

The thin films of polymer-drug were prepared by solvent casting method. The chitosan (2 g) was mixed with 1% acetic acid (100 mL) by constantly stirring at 30 °C for 0.5 h. After obtaining a clear and uniform solution, the pre-weighed drug was added into the chitosan solution and stirred for another 15 minutes. Finally, the polymer-drug mixture was poured into the Petri plate and placed in a vacuum incubator at 30 °C for 24 hours. After the complete drying, the films were cut into 1 × 1 cm size for further analysis. The specific concentration of drug and name of the samples are given in Table 1.

**Table 1:** Composition of drug loaded polymeric thin films

Sample ID	Chitosan (%)	*Cinnamomum verum* (%)	*Angelica sinensis* (%)	*Foeniculum vulgare* (%)	Heparin (%)
C	2	—	—	—	—
CC5		5	—	—	—
CC10		10	—	—	—
CC15		15	—	—	—
CD5		—	5	—	—
CD10		—	10	—	—
CD15		—	15	—	—
CF5		—	—	5	—
CF10		—	—	10	—
CF15		—	—	15	—
CCDF		5	5	5	—
CH		—	—	—	5

### Polymeric matrix degradation studies

The polymer degradation studies were carried out as a function of weight loss over time. The films (1 × 1 cm) were placed in 3 mL of PBS solution at 37 °C. At predetermined time points, the film was taken out, dried and weighed. The weight loss percentage (Δ*W*%) at each time interval was calculated using the eqn (1):^55^1
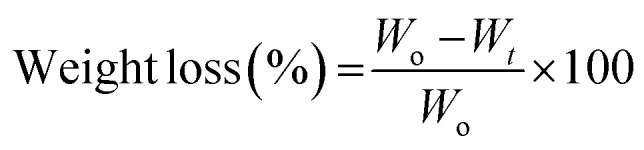
where *W*_o_ is the initial weight of each sample and *W*_*t*_ is the weight of specimen at a specific time interval. All results were estimated from the data of three individual experiments, and the reported data were expressed as mean ± Standard Deviation (SD).

### *In vitro* drug release

For drug release analysis, 1 × 1 cm films were immersed into 3 mL of PBS solution (1 M) at pH 7.4 at 37 °C. After a certain immersion time intervals, the PBS solution was taken out and replaced with fresh PBS solution. The concentration of drug in the solution was measured through UV spectrophotometer. The drug content of *F. vulgare*, *A. sinensis* and *C. verum* was analyzed at 290, 435 and 284 nm wavelength receptively, and eqn (2) was used to calculate the cumulative drug release from the matrix.^56^2
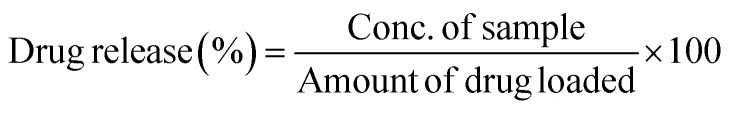


All results were estimated from the data of three individual experiments, and the reported data were expressed as mean ± SD.

### Antioxidant activity evaluation

Antioxidant properties are important for the chelation of free radicals and in this study, DPPH radical scavenging assay was performed for all the samples.^57^ To perform this test, instructions of Gangwar *et al.*, 2014 were followed.^58^ The solution of 2,2-diphenyl-1-picrylhydrazyl (DPPH) was prepared in 0.1 mM methanol. The film samples were immersed in that solution (4 mL) for 60 minutes. After incubation, the UV spectrophotometer at 517 nm wavelength was used to analyze the antioxidative behavior. The eqn (3) was used for analysis.3
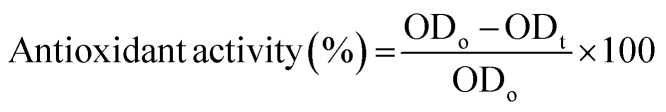
whereas OD_o_ is the absorbance of the control, and OD_t_ is the absorbance of the test samples. Methanolic solution with DPPH was used as blank and ascorbic acid solution was used as positive control.

### Hemolysis evaluation

Hemocompatibility analysis was performed to analyze the effects of medical devices on red blood cells.^59^ For that purpose, the samples were placed in PBS solution (1 M) for 4 hours which was now termed as S1 solution (S1 solution = sample exposed PBS solution). At the same time, 4 mL of PBS solution was added into fresh human blood (2 mL) containing anticoagulants for dilution and centrifuged at 5000 rpm for 5 minutes. The pellet of red blood cells was collected and washed with PBS.

Now, the pellet of red blood cells was exposed to the S1 solution for 4 hours at room temperature. After incubation, the S1 solution was centrifuged at 5500 rpm for 5 minutes. The supernatant was collected for UV-vis spectrophotometry analysis at 540 nm.^56^

The PBS solution was used as blank, and 0.5% Triton X-100 solution was used as positive control. Following eqn (4) was used for calculation.^60^4
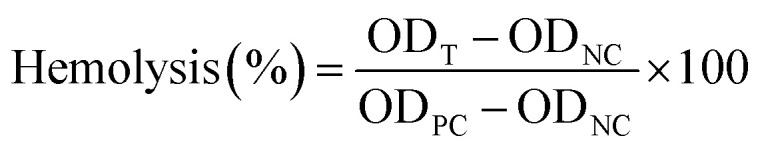
where OD_T_ = OD of test specimen, OD_NC_ = OD of negative control and OD_PC_ = OD of positive control.

### Blood coagulation analysis

For anticoagulation testing, two different tests were performed: plasma thrombin (PT) test and Activated Partial Thromboplastin Clotting Time (APTT).

For PT test, platelet-poor plasma was obtained by the centrifugation of fresh blood at 2500 rpm for 10 min. Now, the samples were placed in Thrombolyzer (Behnk Elektronik GmbH & Co. Germany) along with 1 mL plasma and PT reagent (CaCl_2_ + plasmathrombin) at 30 min at 37 °C.^61^ The machine automatically measured the PT time and gave a reading on the machine display.

Similar process was repeated for APTT test in which APTT reagent was used instead of PT reagent.^62,63^ The APPT time was displayed on the screen of machine.

### Platelet adhesion test

The whole blood from healthy volunteers was obtained after acquiring their consent. All the experiments were performed under the guidelines of the School of Mechanical and Manufacturing Engineering, NUST, Islamabad. The blood was centrifuged for 10 min at 1000 RMP to acquire platelet rich plasma. The pellet was discarded, and supernatant was collected. The films were washed with PBS twice and positioned on 24-well plate. The platelet rich plasma (200 μL) was added on each film and incubated for 2 hours at 37 °C. Afterwards, the specimens were washed with PBS to remove the plasma. The films were incubated for 2 hours, and 24 hours by adding 5% and 2.5% Glutaraldehyde onto them, respectively. Subsequently, the ethanolic solution (25%, 50%, 75% and 100%) was applied for dehydration by successive washing. After drying, films were observed SEM and micrographs were obtained.^64,65^

### Molecular docking

After confirming the coagulation potential of the drug loaded films, molecular docking was performed to observe the interaction and relationship between drug components and proteins of coagulation cascade. For that purpose, CB-DOCK wen server was used and rigid docking approach was utilized.^66^ This is a popular approach which helps in characterization and exhibition of binding of drug molecules at the binding sites of proteins which results in the inhibition of the coagulation cascade.

The structural data of anticoagulation proteins including thrombin (3u69), factor V(7kve), factor VIIa (1qfk) and factor IXa (6mv4) were downloaded from Worldwide Protein Data Bank^67^ and saved in format *.pdb ready for docking simulation. The structures of drug ligands were downloaded from PubChem database and saved in format *.sdf.

The structure of proteins and ligands were submitted to CB DOCK and predicted poses were retrieved. For the selection of best pose and interaction, the values of free energy were utilized. For the analysis of drug protein interaction PyMol (The PyMOL Molecular Graphics System, Version 2.0 Schrödinger, LLC) and LigPlot^++^^68^ were used. For the 3D visualization of macromolecules, PyMole and 2D ligand–protein interaction LigPlot^++^ was employed.^9^ In addition, ligand conformation and orientation in its inhibited-protein active site was visualized on 2D planes.^69–71^

### GC-MS analysis

After confirmation of anticoagulation properties of drug components, *in silico* modelling was performed to find the potential coagulation cascade inhibition method. The objective of GC-MS analysis was to get the confirmation of presence of potential anticoagulation components in the drug extract. For that purpose, N6480013 GC-MS PerkinElmer Clarus 590 GC instrument coupled with a PerkinElmer Clarus SQ 8 S mass spectrometer was used. The method of analysis was developed during our previous studies.^9^ Briefly, samples were prepared in methanol and conditions were set at 40 °C. After two minutes, the temperature started to increase with a gradual increase rate of 1 °C min^−1^ and reached 220 °C. After 120 seconds, it started to increase until reached 310 °C with a heating rate of 20 °C min^−1^. Now, the conditions were maintained for 180 seconds and afterwards, it started to cool down and temperatures gradually decreased. The system was run at electron energy of 70 eV in EI mode. The chromatograms were obtained, and compounds were identified using spectral database of the National Institute of Standards and Technology (NIST) and already published studies in literature.

### Characterization of films

#### FTIR spectroscopy

Fourier transform infrared spectroscopy (FTIR) spectra was used to characterize chitosan thin films containing varying concentrations of different drugs. The films were placed onto the diamond crystal stage of machine and the analysis was performed within the spectral region of 600 to 4000 cm^−1^. The data was plotted using Origin software.

#### Scanning electron microscopy

The surface analysis of drug loaded films was done using scanning electron microscope (SEM) (Quanta 400, FEI, Eindhoven, Netherlands) at an accelerating voltage of 10 kV. Prior to analysis, the films were cleaned and vacuum dried. The 1 × 1 cross section of films was coated with a thin gold film to make the film conducive.

#### Statistical analysis

The statistical analysis of experimental data was performed using GraphPad Prism 9.0 software (California, USA) and Microsoft Excel. For all groups, the experiments were iterated at least three times to get the mean and standard deviations. For further group comparisons were carried out using the two-way ANOVA for finding significance between the means of all possible pairs.

## Results and discussion

### Morphological and structural analysis of thin films

**Fig. 1:**
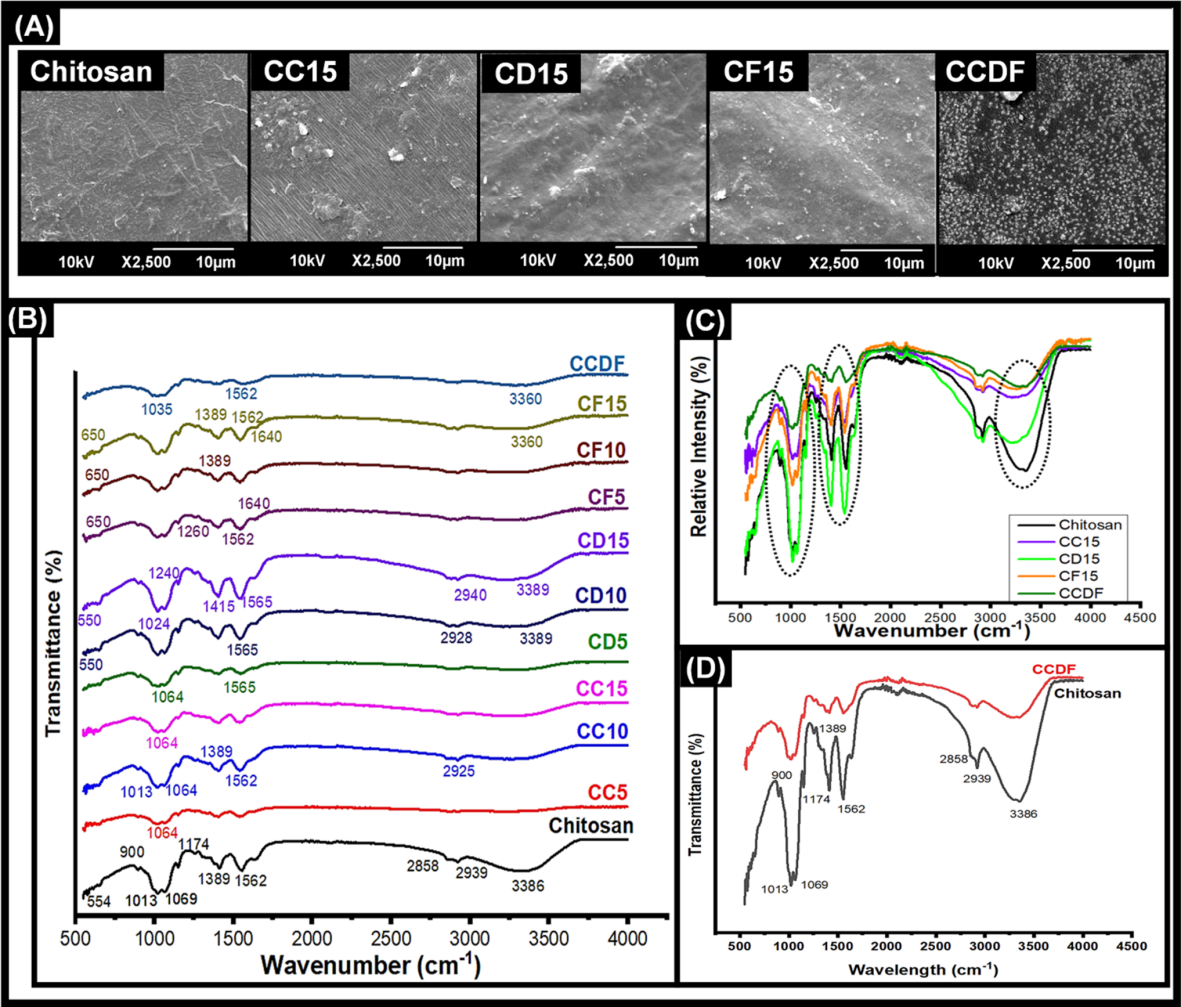
Morphological and structural analysis: (A) SEM analysis of thin films indicating the presence of drugs molecules on the surface (B) FTIR analysis of all the specimens showing variations in intensities of different samples, and emergence and disappearance of peaks. (C) FTIR spectrum showing effects of different drugs on the peak intensities. (D) Comparison of peaks between bare chitosan and CCDF specimen.

The morphology of thin films was observed through SEM micrographs which were presented in Fig. 1. In Fig. 1(A), neat chitosan films demonstrated a smooth surface which was devoid of any cracks and pores indicating good encapsulation and adherence capabilities of chitosan. Similar morphology was shown in a study carried out by Cui *et al.* for the understanding of chitosan film fabrication through novel solvent system.^72^ The surface morphology of thin films modified after the encapsulation of drugs into the polymeric matrix of chitosan.

The films loaded with drugs demonstrated a rough surface having drug particles on the surface. To explain briefly, the *C. verum* loaded film (CC15) showed the presence of a few drug particles on the surface. In addition, the surface seemed rougher as compared to bare chitosan film which may be an indication of a structural change caused by drug loading in the polymeric chains of the matrix. In literature, it was observed that addition of cinnamon essential oil in the chitosan matrix resulted in emergence of pore and holes;^73,74^ however, in this study a lack of porous surface was observed which indicated that drug particles only integrated themselves inside the matrix and did not damage the surface of films. Therefore, it can be speculated that pure extract of *C. verum* had better compatibility with chitosan matrix as compared to its oil-based emulsions.^75^

Similarly, *F*. *vulgare* and *A. sinensis* loaded films CD15 and CF15, respectively flaunted the presence of drug particles on the surface as well as inside the polymeric matrix. The particles on the surface of CD15 had small, round and protruding shape which were similar to the appearance of particles of pure *A. sinensis* described by Wu *et al.*^76^ From literature, it was found that the essential oils of *F*. *vulgare* were not compatible with chitosan-based films because it damaged the surface and made small pores.^77^ The reason could be the weakening of the cohesive forces of matrix due to oil-based formulations of essential oils; however, these challenges were not posed by extracts of drugs. In this study, the use of pure *F*. *vulgare* extract did not pose any harmful effects which could compromise the integrity of the matrix; therefore, matrix retained its flat surface.

The morphology of CCDF was unexpected as a stark difference was found between the appearance of neat Chitosan films and CCDF films as shown in Fig. 1. The CCDF films had a granny appearance similar to the appearance reported by Ashvini *et al.* and Čalija *et al.* which indicated presence of high amount of drug on the surface.^78,79^ Although CCDF had 15% drug concentration just like other thin film samples (CF15, CD15 and CC15), the amount of drug on CCDF was far more than the amount present on other thin films. In addition, the appearance of CCDF was also different from other individual compositions and it was speculated that the affinity of drugs with the matrix decreased upon incorporating them in the form of composite. This can cause an initial burst release and then a sustained drug release kinetics.

After evaluating the morphology of thin films, compositional analysis was performed to map out the drug–polymeric interactions. First, FTIR spectrum for each thin film was obtained and compared with each other in Fig. 1(B). This figure explains the effects of different drugs and their concentrations on polymeric matrix and demonstrated the characteristic bands of chitosan in the frequency range between 4000 and 400 cm^−1^.

Upon the observation of neat chitosan film, the presence of sharp peaks was confirmed at 564 cm^−1^ (out-of-plane bending NH, out-of-plane bending C–O), 901 cm^−1^ (amines), 1013 cm^−1^ (C–N stretch), 1069 cm^−1^ (C–O stretching), 1174 cm^−1^ (stretching of the C–O–C), 1389 cm^−1^ (amide III region), 1410 cm^−1^ (C–C stretch), 1562 cm^−1^ (amide II, stretching vibrations of C–O), 1656 cm^−1^ (C=C stretch), 2149 cm^−1^, 2858 cm^−1^, 2939 cm^−1^ and 3386 cm^−1^ (dimeric OH stretch).^80–83^

The peaks at 1013 cm^−1^ and 1069 cm^−1^ indicating C–N stretch, C–O stretching, respectively was present in all the compositions; however, their intensity decreased by the addition of drugs (Fig. 1(B)) and in composite of drugs, it was almost flattened. The intensity of peaks at 2930 and 2863 cm^−1^ in chitosan remained intact in CD15 composition and reduced in all the other compositions; nevertheless, it disappeared completely in the composite. As these peaks indicated dimeric OH stretching, the reduction in intensity may show the hydrogen bonding between functional groups of drugs and NH and OH groups of chitosan. In addition, same pattern was observed at peaks between 3000 to 3500 cm^−1^ which represented the presence of free hydroxyl groups.^84^

By looking at individual compositions of drugs, it was found that *F. vulgare*-chitosan films demonstrated an increase in peak intensity by increasing the *F. vulgare* concentration. The peaks at 650, 1260 and 1640 indicated the presence of anethol (which is major constituent of *F. vulgare*) and other phenolic groups of *F. vulgare*.^83^ In addition, J. Hussein, Hadi, & Hameed, 2016 proved the presence of aliphatic fluoro compounds at 1029 cm^−1^, alcohols at 1244 cm^−1^ and phenols and hydroxyl groups at 3244, 3275 and 3361 cm^−1^ in *F. vulgare*.^85^ Furthermore, the FTIR spectra of each film containing *F. vulgare* were similar in composition and no chemical reaction occurred that produced new substances. The results of FTIR spectroscopy explained the intermolecular interaction and molecular compatibility between the functional groups in *F. vulgare* and hydroxyl and amino groups in the chitosan chain.

The *A. sinensis* and *C. verum* loaded films at varying concentration showed similar patterns to the *F. vulgare* loaded films. The spectra demonstrated an increase in the intensity of peaks with an increase in concentration of *A. sinensis* in the chitosan matrix. The intensity of bands at 1004–1050 cm^−1^, 1400–1600 cm^−1^ and 2800–3000 cm^−1^ increased and these peaks exhibit the presence of pyranose monomers, carboxylic group and C–H asymmetric stretching vibration of the methyl group, respectively.^86^ Above 3000 cm^−1^, lesser peak intensity identified the decreased number of free –OH groups proving the string hydrogen bonding between *A. sinensis* and chitosan.

In Fig. 1(C), the effects of loading of different drugs in chitosan matrix were observed. It was found that addition of *A. sinensis* had a negligible impact on the intestines of the chitosan spectrum showing a weakest bonding between *A. sinensis* and chitosan. In comparison, *F. vulgare* and *C. verum* showed a significant impact on the composition of chitosan and a prominent decrease in intensities of chitosan matrix was observed. It showed that both *C. verum* and *F. vulgare* had a strong compatibility with chitosan matrix. A particularly conspicuous decrease in peak intensities was observed in CCDF specimen as the interaction between drug molecules and chitosan matrix was so strong that it flattened many peaks. It also proves the compatibility of all three drug molecules with each other and with polymeric matrix.

The difference between the compositions of neat chitosan film and CCDF specimen is shown in Fig. 1(D). It showed a sharp decrease in the peak intensities of polymeric matrix after addition of the drug extracts. It also showed a strong interaction between drug particles and polymeric matrix which ensured that drug was not just loosely present on the surface (as shown in SEM analysis) but also embedded inside the matrix and was interacted with polymeric chains.

Overall, the morphological and structural analysis showed that drug molecules not only attached themselves on the surface of the thin films but also interacted with polymeric chains of the matrix and made hydrogen bonding. This kind of interaction helps in an initial burst release followed by a sustained release drug kinetics. In addition, no new peaks emerged in spectrum indicating intact chemical structure of chitosan matrix and absence of any compositional modifications.

### Polymer degradation and drug release studies

**Fig. 2:**
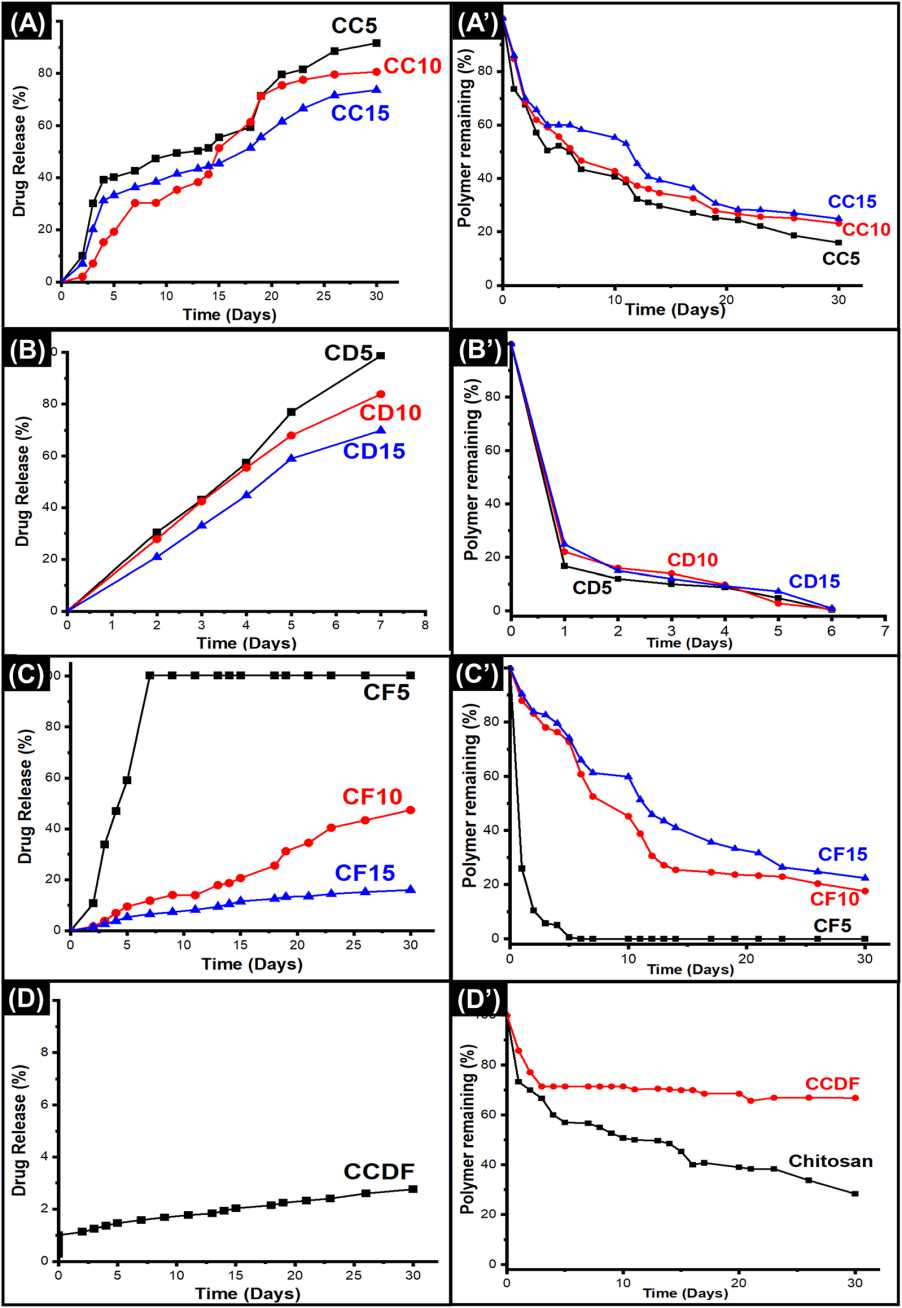
Drug release and polymer degradation of all the compositions indicating that addition of different concentrations of drug into the matrix affects the degradation and drug release patterns. (A) Drug release kinetics of *C. verum* loaded thin films, (B) drug release from *A. sinensis* showing fastest drug release kinetics, (C) drug release pattern of *F. vulgare* over 30 days, (D) demonstration of effects of combining all three drugs into one matrix, (A’) polymer degradation patterns of different *C. verum* loaded thin films, (B’) degradation rate of *A. sinensis* loaded thin films, (C’) degradation of *F. vulgare* loaded chitosan matrix over 30 days, (D’) degradation pattern of CCDF matrix containing all three drugs.

The degradation rate and effects of drug concentration on chitosan degradation were mapped through gravimetric method and results were presented in Fig. 2(A’)–(D’). This analysis was performed for all the thin films to observe the effects of different drugs with varying concentrations on polymer degradation and release kinetics.

In Fig. 2(D’), it was observed that pure chitosan lost more than 20% matrix in 24 hours and after that the rate of degradation was slowed down and roughly 70% mass was lost in 30 days. The degradation pattern of chitosan matrix was just like one reported by Bryuzgina *et al.* for chitosan films.^87^ The initial degradation was attributed to the hydrolysis of polymeric chains which opened the cavities on the surface of thin films and lost mass. With the passage of time, these cavities kept increasing in size and number which caused the loss of structural integrity of films leading towards the complete degradation of matrix.

It was observed that the incorporation of drug extracts into matrix affected the degradation rates of the chitosan films. As shown in Fig. 2, among all the compositions, *A. sinensis* loaded films had the most obvious effects on the chitosan matrix as it degraded the whole matrix within 5 days. Overall degradation rate was *A. sinensis* > *F*. *vulgare* > *C. verum* > composite.

The patterns of degradation of *C. verum* loaded films remained the same for all three compositions; however, it affected the rate of weight loss as shown in Fig. 2(A’). The CC5 exhibited the fastest polymer degradation rate indicating 84% weight loss in 30 days. The rate of weight loss reduced when the concentration of drugs was increased in CC10 and CC15 specimens as the depicted 77% and 75% degradation in a month. The results indicated that addition of *C. verum* loosened the chains of polymeric matrix and occupied the new gaps in the matrix; thus, it made the film vulnerable to hydrolytic degradation. Nevertheless, increase in drug concentration decreased the degradation of matrix. The reason may be that at higher drug concentrations, the volume of drug particles occupying open spaces inside matrix increased which reduced the catalytic sites for hydrolysis resulting in a sturdier specimen that resisted degradation for longer period of time.^88^

The *F. vulgare* loaded films (CF5, CF10, CF15) demonstrated degradation patterns similar to the patterns of *C. verum* loaded films as presented in Fig. 2(C’). The sequence of degradation was CF5 > CF10 > CF15. The CF5 films degraded within 5 days demonstrating fastest weight loss among all the specimens. In a study, Yulizar *et al.* reported the catalytic activity of *F. vulgare* in a nanocomposite which promoted the cleavage of the polymeric chains of drug carrier.^89^ This activity may have role in unstableness of the chitosan matrix in CF5 specimen which resulted in complete loss of weight of polymeric films within 5 days. The remaining compositions CF10 and CF15 demonstrated that only 17% and 22% matrix remained respectively, after 30 days which was similar to *C. verum* loaded films.

Surprisingly, the situation was different for *A. sinensis* loaded films which exhibited 85%, 78% and 76% matrix degradation after 24 hours for CD5, CD10 and CD15 specimens, respectively (Fig. 2(B’)). The *A. sinensis* is rich in polysaccharides and other components which may have interfered with the three-dimensional polymeric structure of chitosan resulting in weakening of intermolecular forces and degradation of matrix. The ions and drug components released from *A. sinensis* initiated the drug-mediated hydrolysis of matrix and caused the scission of polymeric chains.^90^

The results of CCDF degradation (Fig. 2(D’)) revealed slower weight loss of chitosan matrix. The results demonstrated that 15% and 23% CCDF matrix was degraded in 24 hours and 48 hours, respectively. It was observed that the rate of matrix degradation became slower after 2 days and only 30% and 34% matrix could be degraded within 15 and 30 days, respectively. These results indicated that the composite of three drugs had strong affinity with the polymeric chains of matrix and protected the cleavage side from the attacks of ions or other catalytic moieties. Secondly, FTIR analysis in Fig. 1(D) showed that the intermolecular forces *i.e.*, covalent bonds, H-bonds and van der Waals forces were stronger between chitosan and drug molecules as compared to intramolecular forces of chitosan which resulted in slower degradation rates of CCDF *versus* neat chitosan matrix.

If we compare these results with commercially available implants, this degradation rate is comparable to Orsiro cardiac implant [Biotronik Germany] and Ultimaster cardiac implant [Terumo, Japan].^36^ Clinical studies have shown that the Orsiro implant system utilized PLA material as a drug carrier. This PLA material underwent a slow degradation, with approximately 6% degrading within the first 30 days. Like the Orsiro implant, Terumo exhibited a degradation profile where roughly 20% degraded within the first 30 days, with complete degradation in 150 days.^91^ The results of CCDF are little faster than these systems as roughly 34% matrix was degraded in 30 days which shows that CCDF system holds promise as a viable drug delivery platform.

The drug release of polymeric matrix greatly depends upon the degradation rate and affinity between drug and polymer. The effects of different concentrations of drug and different types of drugs on the release kinetics had been observed in this study and presented in Fig. 2(A)–(D). The results indicated that all compositions underwent an initial burst release which was followed by a sustained drug release from the matrix. It was found that varying the drug type affected the release kinetics differently and overall pattern of drug release was *A. sinensis* > *C. verum* > *F*. *vulgare* > composite.

As shown in Fig. 2(A), the *C. verum* loaded films had intermediate drug release kinetics among other compositions and by increasing the amount of drug in the matrix, the release became slower. The trend of *C. verum* release from matrix was CC5 > CC10 > CC15. The drug release kinetics were heavily dependent upon the polymer degradation rate because hydrolytic cleavage of chains generated free spaces and voids that opened up the drug pockets inside the matrix and released it into the solution. Therefore, polymeric matrix of CC5, CC10 and CC15 degraded 27%, 16% and 14%, respectively within 24 hours, 5%, 1% and 3% drug was released. After one month, the remaining drug in the matrix was 9%, 20% and 27%, respectively.

The *A. sinensis* loaded matrix demonstrated unusual drug release pattern (Fig. 1(B)) as compared to the drug release kinetics of other drugs; however, it was translated according to the degradation of matrix exhibited in Fig. 2(B’). The CD5 specimen released 100% drug within 7 days which was due to complete degradation of polymeric matrix containing the drug. The other two compositions released 84% and 70% drug within 30 days. Although the matrix was completely dissolved, the remaining drug could not be detected in the solution. The reason may be the strong attachment of *A. sinensis* particles with polymeric broken chains which did not allow the release of drug into the solution even after the matrix degradation. At lower concentration, *F. vulgare* loaded films (CF5) degraded fast and released all the drug within a short span of time as shown in Fig. 2(C) and (C’); however, the CF10 and CF15 specimens exhibited a steady drug release up to 50% in 30 days.

When the drugs were combined and incorporated into the matrix (CCDF), it not only slowed down the rate of polymer degradation but also made the matrix strong to hold the drug for longer period of time as presented in Fig. 2(D). There was an initial burst release within 24 hours which was followed by the sustained drug release pattern. Looking at the drug release patterns of commercially available drug eluting coronary stents, it was found that initial burst release of drug followed by a sustained drug release is necessary for appropriate healing of lesion at target site.^36^ The reason is that when medical devices are implanted inside human body, these create a lesion at target side to open the blocked area; hence, creating a lesion at the site which invites a plethora of platelets and growth factors to heal the lesion and can trigger the coagulation cascade leading to the early thrombus formation and device failure.^92^ If an anticoagulant is coated on the medical device, it will be able to suppress the early thrombus formation through burst release of drug and once critical window is passed, it will keep releasing the drug in sustained manner.

In conclusion, chitosan-based drug loaded matrix demonstrated appropriately slower degradation rate as compared other polymers including poly lactic acid and poly vinyl school which made it suitable for drug coated biomedical implants. In addition, increasing the drug in the matrix slowed down the degradation rate. The free drug particles on the surface of matrix not only cause the initial burst release but also their removal from the surface expose the matrix to external environment and give an opportunity to water molecules to seep inside matrix and accelerate the degradation pattern. Furthermore, the degradation of matrix creates voids and spaces in the matrix which further increases the degradation rate. The results of chitosan-based matrixes were a bit surprising due to strong interaction between drug and protein molecules which caused a very slow release of drug from the matrix.

### Antioxidant activity evaluation

**Fig. 3:**
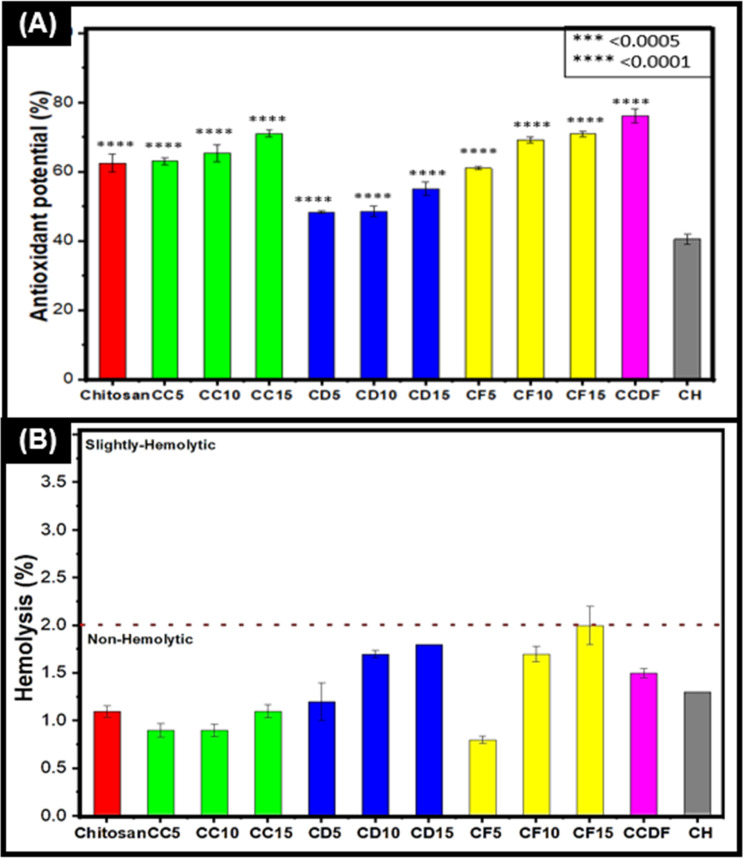
Antioxidant, anticoagulation and biocompatibility related analysis for thin films. (A) Antioxidant potential of films was increased by increasing the drug concentration. (B) All the films were hemocompatible but the hemocompatibility character was increased by decreasing the drug concentration. All the experiments were reiterated 3 to 5 times to obtain mean values and error bars represent SD.

The antioxidant potential and ROS scavenging ability of drug loaded films were evaluated through DPPH radical scavenging test. The results presented in Fig. 3(A) showed that all the films possessed antioxidant properties and drug loading enhanced the scavenging ability of the chitosan and the order was CCDF > CF15 > CC15 > CF10> CC10 > CC5 > C > CF5 > CD15 > CD5 > CD10 > CD5 > CH and it revealed that *F. vulgare* and *A. sinensis* had highest and lowest antioxidation properties, respectively.

All the films exhibited a significant scavenging ability in a dose dependent manner. To explain further, the films of *F. vulgare*, CF15, CF10 and CF5, demonstrated the highest ability of ROS scavenging which increased in ascending manner along with drug concentration *i.e.*, 61, 69.2 and 70.9, respectively. CF15 had 70.9% (*p* = 0.0001) antioxidant activity and it was significantly better than heparin loaded films which were used as experimental control throughout the study. The higher antioxidative properties may be associated with the presence of phenolic compounds and *trans*-anethole in *F. vulgare*. In addition, the increase in *F. vulgare* concentration in matrix may increase the concentration of antioxidative compounds which ultimately enhanced the antioxidation properties. These results were in line with the findings of Oktay *et al.* who reported up to 99% of antioxidant activity of *F. vulgare* and showed that its free radical scavenging activity increased with increasing drug concentration.^93^

The thin films loaded with *A. sinensis* showed significant antioxidation capability [CD5 = 48.3% (*p* = 0.0001), CD10 = 48.6 (*p* = 0.0001) and CD15 = 50% (*p* = 0.0001)]; however, it was the lowest among other drug loaded thin films. *A. sinensis* had phenols, ferulic acid and caffeic acid that were responsible for the antioxidation properties.^94^ In a study, Huang *et al.* demonstrated a positive relationship between phenols and antioxidant properties of *A. sinensis*;^95^ nevertheless the processing conditions (like time or ethanol concentration) during extraction may affect the antioxidation capabilities.

It is notable that chitosan itself has good antioxidation capacities (62%) and literature has suggested replacing the synthetic antioxidants with chitosan-based material [580, 581]. In this study, the antioxidation abilities of drug loaded chitosan matrix were elevated which were observed on all films particularly in consortium of different drugs (CCDF) as it demonstrated 76.1% (*p* = 0.0001) antioxidation capability. In contrast, loading of heparin into chitosan decreased (40% *p* = 0.0001) the antioxidation capabilities. The antioxidative capabilities of heparin are debatable as Lapenna *et al.* and Mohanty *et al.* had reported that heparin did not possess the antioxidation capabilities.^96,97^ Compared to CCDF and other specimens, commercially available synthetic anticoagulants heparin and aspirin exhibited lower antioxidant potential. Oliveira *et al.*^98^ reported that aspirin demonstrated less than 20% antioxidant activity as measured by the DPPH radical scavenging capacity assay. In contrast, CCDF specimen demonstrated more than 70% antioxidative potential. Higher antioxidative potential helps to neutralize ROS and reduce oxidative stress which ultimately lessens the inflammatory cascades. It also enhances the biocompatibility of materials and in case of bone implants, it reduces periprosthetic osteolysis and promote bone healing.

Overall, CCDF showed excellent antioxidation properties as compared to synthetic drug in this study and ability to protect the body from harmful effects of ROS species during thrombo-inflammatory reactions.

### Hemolysis evaluation

The percentage of hemolysis was measured to analyze the effects of drugs on red blood cells (RBCs) and the results were presented in Fig. 3(B) which indicated that all the drug compositions as per criterion of ASTM F756 standard were non-hemolytic. It was observed that concentration of drug was an important factor which affected the hemolysis rate of specimens. The rate of hemolysis was linearly increasing with the drug concentration. To explain briefly, the results indicated that *C. verum* had lowest hemolytic potential among all three drugs. The trend of hemolysis among different compositions was *F. vulgare* > *A. sinensis* > Composite > *C. verum*.

The highest hemolysis potential (2%) was demonstrated by CF15 specimen; however, this value comes under the range of non-hemolytic materials. The lowest hemolytic potential was shown by CF5 which shows that lesser concentrations of *F. vulgare* does not cause the disruption of RBCs; however, the increasing concentrations have effects on the rupture of RBCs. These results were in agreement with Sharopov *et al.* who reported that the essential oil of *F. vulgare* can lyse the membranes of cells at higher concentrations. At lower concentration, it does not have any harmful effects on blood cells.^99^ Similarly, Qui *et al.* described the non-hemolytic behavior of fennel oils in literature and proved the safety of *F. vulgare* for medical applications.^100^

The hemolytic potential of pure chitosan was 1.1% (as shown in Fig. S6 in ESI†) which was not affected by the addition of *C. verum*; however, it increased to 1.2%, 1.7% and 1.8% by the incorporation of 5%, 10% and 15% *A. sinensis* into the matrix (CD5, CD10, and CD15 respectively). In literature, Salehi *et al.* provided results similar to this study and proved that increasing concentration of *C. verum* can enhance hemolytic potential, but it was not significant.^101^ López-Mata *et al.* provided the insights about cinnamon loaded chitosan films and found them non-hemolytic.^102^ Chang *et al.* evaluated the effects of ethanolic AS extract on blood parameters of rats and found it to be a promising therapeutic candidate for biomedical applications.^103^ The consortium of the drugs (CCDF) also demonstrated a non-hemolytic behavior by showing 1.5% blood hemolysis. In conclusion, all the drugs either individually or in the composite form were non-hemolytic and potentially invulnerable for biomedical applications.

According to ISO standard 10993, determining the biocompatibility of medical implants is necessary and one crucial test mandated by this standard is the evaluation of hemolytic potential of the implant. All the specimens in this study had non-hemolytic potential just like the results demonstrated by Kuznetsov *et al.*^104^ when they tested their biomedical implant specimens in rabbit iliac artery. Reassuringly, similar results were observed when Meng *et al.*^105^ evaluated the hemolytic potential of magnesium alloy, a promising material currently under investigation for erodible biomedical implants. The concordance of results of this test with prior research from literature suggests that specimens of this study possess promising qualities for biomedical implant applications.

### Anticoagulation behavior of films

To analyze the anticoagulation ability of drug loaded thin films, PT and APTT tests were performed. Both tests gave insights about the possible mechanism of blocking of coagulation cascade of drugs. The anticoagulation potential of all the compositions is given in Table 2.

**Table 2:** Anticoagulant potential of drug loaded thin films

Specimen ID	APTT	PT	INR
Chitosan	31 ± 0.4	14.5 ± 0.4	1.1
CC5	30 ± 0.1	14.6 ± 0.1	1.1
CC10	30 ± 0.3	14.8 ± 0.3	1.2
CC15	30.9 ± 0.1	15 ± 0.1	1.2
CD5	30.2 ± 0.3	14.6 ± 0.3	1.1
CD10	30.2 ± 0.1	15 ± 0.1	1.2
CD15	40.7 ± 0.2	15.5 ± 0.2	1.2
CF5	30.1 ± 0.1	14.5 ± 0.1	1.1
CF10	30.2 ± 0.1	14.8 ± 0.1	1.2
CF15	31.1 ± 1.1	15.2 ± 1.1	1.2
CCDF	30	135	13.1
CH	135	45.8 ± 1	3.99

The results of the anticoagulation test showed that all the thin film samples have anticoagulation activity. Among all the drugs, *A. sinensis* had the highest ability of blocking coagulation cascade and could delay the blood coagulation more than two seconds (15.5 s) as compared to normal blood coagulation (13 s). Also, the anticoagulation potential of directly related to the concentration of drug loading in films as CD15 exhibited highest anticoagulation activity. These results are in line with the findings reported by Lee *et al.* in a clinical study in which anticoagulation potential of *A. sinensis* was reported along with a few other herbs was evaluated in 75 patients^106^ and results showed that *A. sinensis* has a significant impact on anticoagulation of blood and even found to reduce the epinephrine-induced aggregation in 2 subjects. In another study, Wang *et al.* analyzed the constitutes of *A. sinensis* and reported that its anticoagulation activity potential comes from the constitutes from petroleum ether-soluble fraction. Upon further analysis, 26 components were found which belonged to phenol acids, the phthalides, and the aliphatic acids and among these components, phthalic acid, vanillic acid, ferulic acid, senkyunolide I, butylphthalide, ligustilide, linolenic acid, senkyunolide D and senkyunolide F were majorly responsible for the anticoagulation activity.^107^

The *F. vulgare* also demonstrated mild anticoagulation potential as it was able to delay the blood coagulation by a few seconds in both PT and APTT test (described in Table 2). The trend of anticoagulation activity was directly related to the concentration of drug loaded in thin films as CF5 was able to delay the coagulation time by 2.5 seconds in comparison to anticoagulation potential of CF15 which delayed the coagulation time by 3.2 seconds. In literature, anticoagulation effects of *F. vulgare* have already been proven through different clinical studies in which a delay in coagulation time was observed after orally taking *F. vulgare*.^108^ Even Farid *et al.* reported immune enhancement, and antimutagenic efficacy of *F. vulgare* by using human blood cultures.^109^

The anticoagulation potential *C. verum* was similar to other two drugs as it also demonstrated an increase the anticoagulation activity with an increase in the drug concentration in the polymeric matrix. Cinnamon has been historically famous herbal medicine and an anti-inflammatory dietary supplement which has proven antidiabetic,^110^ anti-inflammatory^111^ and anticoagulant properties^9^ due presence of components like cinnamaldehyde, coumarin, limonene, α-terpineol and many others.^112^ In a study, antithrombotic and antiplatelet effects of plant-derived compounds were analyzed by Kubatka *et al.* and it was reported that cinnamic acid which is an essential component of cinnamon has a strong ability of anticoagulation^113^ and could delay the blood coagulation time by 18.2 s.^114^ In our study, it was able to delay the coagulation time by a few seconds and it could be retardation of the activity could be related to drug polymer relationship as embedding a drug in polymeric matrix may affect the pharmacological activities of drug components. Overall, all the polymeric matrixes loaded with *C. verum* demonstrated mild anticoagulation activities.

When all the drugs were combined in single matrix (CCDF), a sudden rise in anticoagulation activities was observed. For individual drugs, the coagulation time was prolonged for only a few seconds; however, the consortium of drugs prolonged the time for more than 130 seconds. This phenomenon was surprising; nevertheless, in depth deliberation showed that all three drugs were compatible each other and instead of affecting each other's structure and functions, these complemented each other and enhanced the functionality. We also found the evidence from literature that one of drug component *A. sinensis* does not have a strong anticoagulation potential; however, when combined with other anticoagulants, it significantly enhances the overall activity of the drugs.^115^ This phenomenon is line with herb–herb combination theory and multi-item concoction which explained that factors like synergistic multi-target effects receptors and proteins, change in pharmacokinetic or physiochemical effects based on improved solubility, resorption rate, and enhanced bioavailability and neutralization of adverse effects other substances present in the mixture may be associated with enhanced activity of herbs in a combined dosage form.^116^

In this study both PT and APTT analysis were performed and interestingly, the individual thin films loaded with *A. sinensis* showed a gradual increase in anticoagulation activity in PT test with increasing the drug concentration in the matrix; however, no activity was observed in lower concentrations of drug during APTT testing and only CD15 showed an anticoagulant activity. It is known that drugs can inhibit the coagulation cascade either by blocking extrinsic pathway or intrinsic pathway. The lack of activity during APTT testing showed that these drug molecules are unable to affect the proteins of intrinsic pathway (factors XII, XI, IX, and VIII). Similar results were found in other individual drug-polymer compositions; however, CCDF specimen did not show an activity in APTT analysis and a high activity in PT analysis exhibiting a lack of interaction with proteins of intrinsic pathway and a strong relationship with proteins of extrinsic pathway and vis versa was true for heparin loaded polymeric matrix. This information was helpful for mapping out the inhibitory pathway for our proposed drug.

The first successful human trials of Palmaz-Schatz coronary stents were coated with the strong anticoagulant heparin^117^ and in our study, natural drugs with potent anticoagulation potential were used because heparin is associated with the risk of spontaneous bleeding and thrombocytopenia. Therefore, Lu *et al.* developed heparin-like anticoagulant polypeptides which had anticoagulative and clot solubilities properties.^118^ A previous study by Ozaltin *et al.*^119^ proposed polyethylene terephthalate (PET) surfaces modified with plasma-immobilized fucoidan as a material for medical implants because this modification extended the PT of surface more than 20 seconds which demonstrated that it gained anticoagulant properties. Our surface exhibited an anticoagulation potential exceeding theirs by more than tenfold. Thus, biomedical implants with anticoagulant properties demonstrably enhance clinical outcomes and device performance.

As CCDF specimen demonstrated best anticoagulation potential among all the specimens, it was selected for further analysis.

### *In silico* modeling

**Fig. 4:**
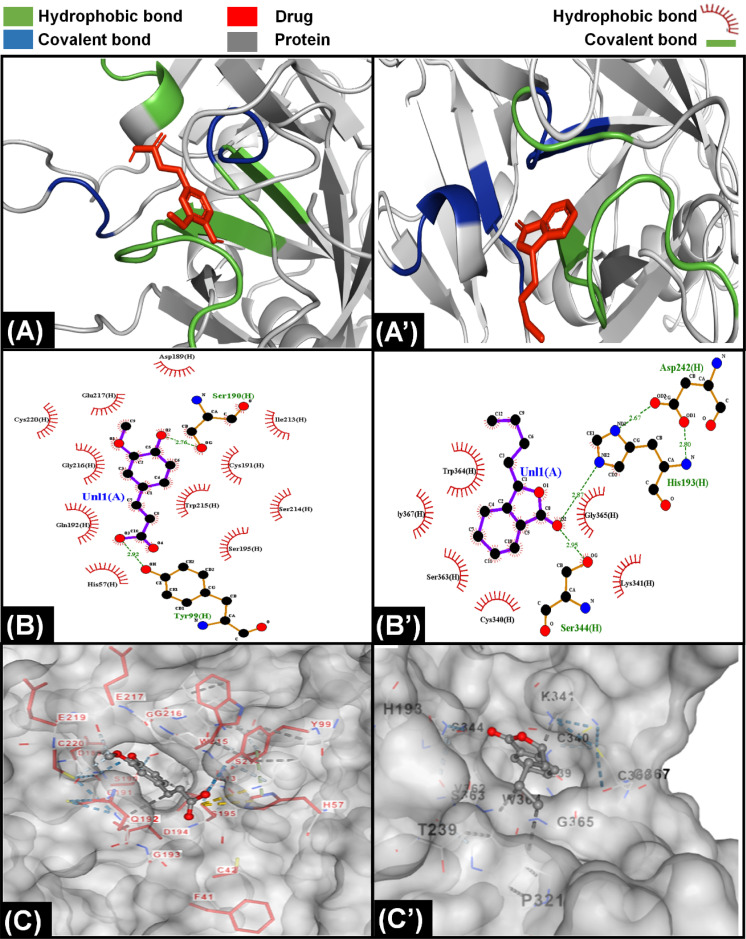
Molecular interaction of components of *A. sinensis* with proteins of extrinsic pathway. (A) Interaction of ferulic acid with protein Xa indicating that the ligand was bound with protein through hydrophobic and covalent interactions. (A’) Interaction of senkyunolide with VIIa showing the presence of both hydrophobic and covalent interaction with protein. (B) LigPlot demonstrating presence of eleven hydrophobic and three covalent bonds between ferulic acid and Xa protein. (B’) LigPlot showing presence of six hydrophobic and three covalent bonds between senkyunolide and VIIa protein. (C) The attachment of ferulic acid inside a cavity present on the surface of Xa protein. (C’) The position of senkyunolide inside a cleft of VIIa protein.

The information from PT and APTT analysis was utilized to map out the possible coagulation cascade inhibition pathway for CCDF specimen. As CCDF did not show any activity during APTT analysis indicating that it is not interacting with factors XII, XI, IX, and VIII and showed a high activity in PT analysis; therefore, the interaction of anticoagulation component of CCDF with factors VII, III, X, V, II, I, and XIII were analyzed and results are showed in Fig. 4.

In literature it was found that components like ferulic acid,^120^ senkyunolide^121^ and phthalide derivatives from *A. sinensis* are responsible for anticoagulation activities of the drug extract; therefore, it was assumed that in CCDF specimen, these two components are playing a role in blocking extrinsic coagulation pathway and their interaction with VII, III, X, V, II, I, and XIII was analyzed. The interaction of ferulic acid with different proteins is demonstrated in results Fig. 4 and ESI Fig. S1.† It was found that ferulic acid had the strongest interaction with Xa protein by making more than 10 hydrophobic bonds and two covalent bonds (Fig. 4(A)–(C)). In comparison, it was making seven hydrophobic and two covalent bonds with factor V and II and nine hydrophobic bonds and one covalent bond with factor VIIa; therefore, it was speculated that ferulic acid was able to block the activity of factor Xa and Fig. 4(C) showed that it was not just a surface level interaction but the drug ligand was able to snuggle inside the cleft of factor Xa and completely block the activity. In the case of senkyunolide, it strongly interacted with VIIA protein through three covenant and more than five hydrophobic bonds (Fig. 4(A’)–(C’)). In ESI figures (Fig. S2†), it was shown that senkyunolide did not make any covalent bond with factor V and II and only made hydrophobic bonds demonstrating a rather week interaction with proteins other than factor VIIa. It was also able to penetrate in a cleft present at the surface of protein to block the activity. Overall, it was suggested that components of *A. sinensis* were able to block the extrinsic coagulation pathway by inhibiting the activity of factor Xa and VIIa.

**Fig. 5:**
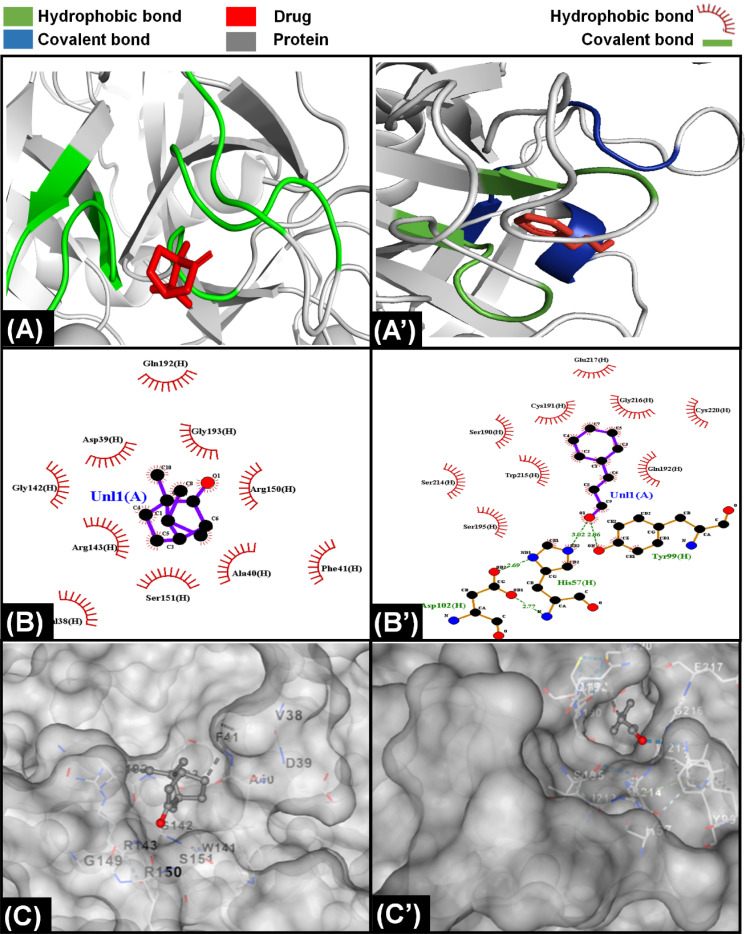
Molecular interaction of components of *C. verum* with proteins of extrinsic pathway. (A) Interaction of camphor with protein Xa indicating that the ligand is bound with only hydrophobic interactions with protein. (A’) Interaction of cinnamaldehyde with Xa showing the presence of both hydrophobic and covalent interaction with protein. (B) LigPlot demonstrating presence of ten hydrophobic bonds between camphor and Xa protein. (B’) LigPlot showing presence of nine hydrophobic and three covalent bonds between cinnamaldehyde and Xa protein. (C) The attachment of camphor on the surface of Xa protein (C’) The position of cinnamaldehyde inside a cleft of Xa protein.

For *C. verum*, it was found from literature that cinnamaldehyde^122^ and camphene^123^ may be responsible for the anticoagulation activities. Their interaction with proteins of extrinsic pathway were analyzed and demonstrated in Fig. 5A, ESI Fig. S3 and S4.†

Camphene is cinnamon polyphenol and its interaction with factor Xa of extrinsic coagulation pathway is depicted in Fig. 5(A)–(C) showing the presence of more than five hydrophobic bonds. Further analysis with other proteins showed that it only made hydrophobic bonds with all the proteins of coagulation cascade and did not make any hydrogen bond. In comparison, cinnamaldehyde strongly interacted with all the proteins as shown in ESI Fig. S4;† however, strongest interaction was observed with factor Xa in Fig. 5(A’)–(C’) by making multiple hydrophobic and covalent bonds. The disparity in interaction of camphene and cinnamaldehyde with factor Xa was also observed in their energy scores which were −4.6 and −5.6 kcal mol^−1^ (described in ESI Table 1†), respectively for both of them showing higher ligand efficiency of cinnamaldehyde. Similar analysis was performed by Khadke *et al.* to observe the interaction of cinnamaldehyde to evaluate its activity as anthelmintic agent.^124^ In conclusion, the components of CCDF coming from *C. verum* were able to block factor Xa which ultimately played a crucial role inhibition of coagulation cascade.

**Fig. 6:**
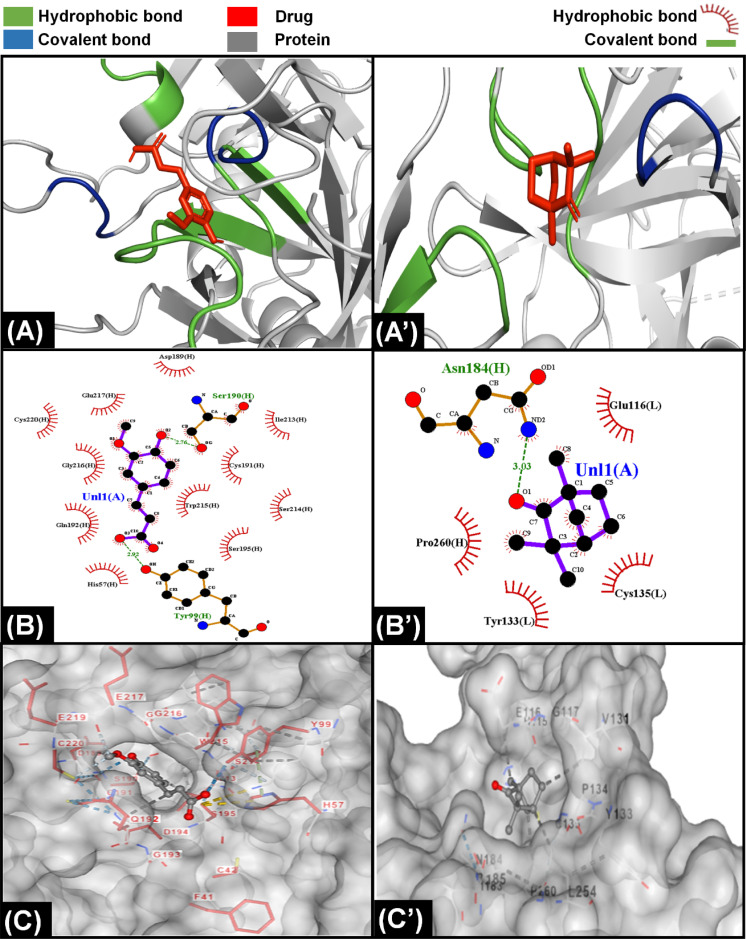
Molecular interaction of components of F. vulgare with proteins of extrinsic pathway. (A) Interaction of ferulic acid with protein Xa indicating that the ligand was bound with protein through hydrophobic and covalent interactions. (A’) Interaction of fenchone with VIIa showing the presence of both hydrophobic and covalent interaction with protein. (B) LigPlot demonstrating presence of eleven hydrophobic and three covalent bonds between ferulic acid and Xa protein. (B’) LigPlot showing presence of four hydrophobic and single covalent bonds between fenchone and VIIa protein. (C) The attachment of ferulic acid inside a cavity present on the surface of Xa protein. (C’) The position of fenchone inside a cleft of present on the surface of VIIa protein.

Throughout history, *F. vulgare* remained an important medicinal plant which had anti-inflammatory, antithrombotic and anticancer properties. It is a rich plant having multiple components; however, ferulic acid and fenchone may be responsible for its anticoagulation properties. The interaction of components of *F. vulgare* with the proteins of extrinsic factor is demonstrated in Fig. 6, ESI Fig. S1 and S5.† Ferulic acid demonstrated a stronger interaction with coagulation proteins as compared to fenchone which is evident from the higher number of hydrogen hydrophobic bonds between drug ligands and proteins. In addition, the ferulic acid ligand was able to penetrate inside a cavity of protein to block its activity; however, fenchone simply snuggled inside a cavity present on the surface of the protein. Overall, both components were able to attack coagulation cascade proteins from multiple sides and inhibit the activity.

As CCDF specimen was a consortium of *C. verum*, *F. vulgare* and *A. sinensis*, it had multiple anticoagulation components which were able to attack multiple proteins of coagulation cascade resulting in blocking the pathway for a longer period of time. This heightened effect had already been proven by the experimental data from PT and APTT testing as CCDF demonstrated a high anticoagulation potential as compared to individual compositions. The prolongation in blood coagulation plays a crucial role because it affects the endothelial process, the inflammatory response, and thrombus formation.^125^

**Fig. 7:**
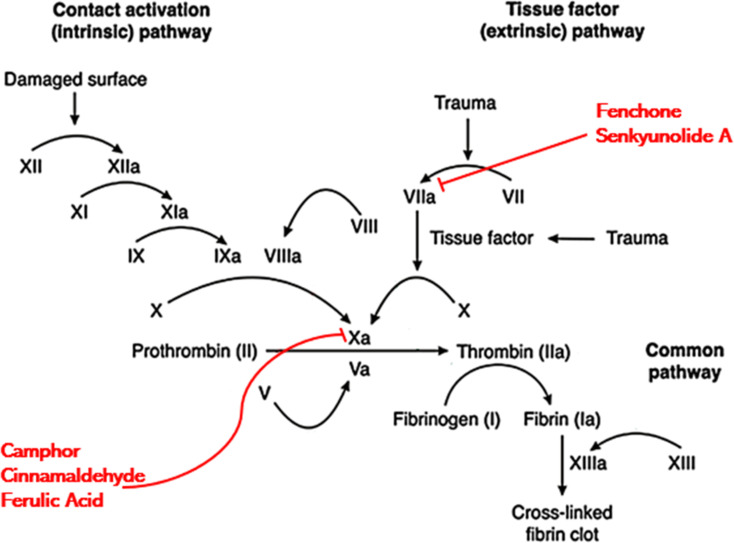
Proposed mechanism of action indicating that different components of CCDF specimen can bind with Xa and VIIa proteins and block them leading to inhibition of coagulation at the target site.

Generally, anticoagulants used in biomedical industry belong to either vitamin K dependent antagonists, low molecular weight heparin, direct thrombin blockers or factor Xa blocker. The drug we are proposing belongs to direct coagulation cascade blockers as it blocked not only factor Xa but also inhibited the activity of other coagulation factors making it a more potent drug. On the basis of results of molecular docking, the potential inhibition pathway for proposed drug is explained in Fig. 7 which indicated that in the CCDF specimen, the ligands bound strongly with Xa and VIIa proteins and blocked the coagulation cascade at these two points.

### Confirmation of presence of anticoagulant components

**Fig. 8:**
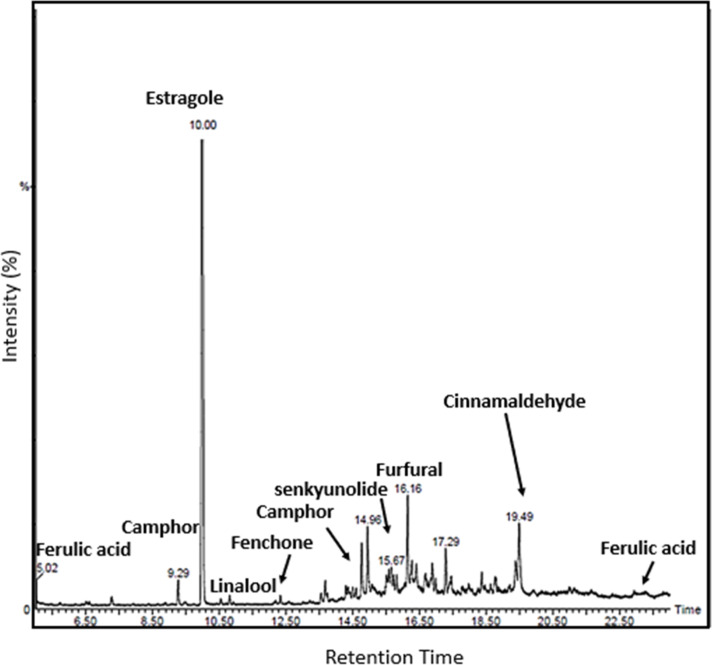
GC-MS analysis of PCGT specimen confirmed the presence of required anticoagulants in the CCDF specimen.

The GC-MS analysis was performed to identify and confirm the presence of anticoagulant components of CCDF specimen and results were exhibited in Fig. 8. The profiles of volatile constituents were identified by comparing mass spectra and retention time based on the standards and research literature. Only those constituents have been mentioned which have anticoagulation properties.

It was known that eugenol, coumarin, camphor, and cinnamaldehyde were part of *C. verum* which might be associated with the strong anticoagulation activities and their presence was identified in the drug extract. Cinnamaldehyde was an important component of *C. verum* and its appearance was identified by comparing the results with GC-MS chromatogram of Friedman *et al.* which was obtained studying the different foods containing cinnamon.^126^ Similarly, ferulic acid, estragole and fenchone were found in *F. vulgare* specimens and senkyunolide was the component of *A. sinensis* and their presence was confirmed through literature^127–133^ and labelled accordingly. Overall, the results of GC-MS are in line with literature and FTIR spectrum of the drug previously described in the study.

### Antiplatelet adhesion potential of films

**Fig. 9:**
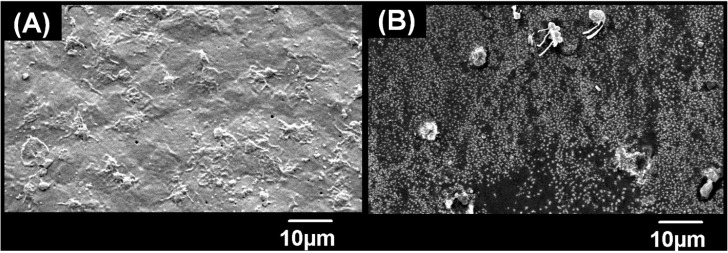
Platelet adhesion test. (A) The platelets were attached to the surface of bare chitosan in large number and formed a complete network on the surface. (B) Only a few platelets could attach to the surface as the presence of drug particles inhibited the attachment of platelets.

The anticoagulation potential of the CCDF was already proven and presence of anticoagulation components was already done. From literature, it was found that anticoagulation potential of specimens reduces the rate of platelet adhesion to the surface which ultimately plays a role in reduction of thrombus formation at the target site. The objective of this test was to analyze the platelet inhibition potential of the CCDF specimen and compare it with inhibition potential of bare chitosan. The results were presented in Fig. 9 which showed that the ability of platelet attachment to the surface of thin films greatly reduced by the addition of drugs. The qualitative SEM analysis demonstrated the presence of a large number of platelets on the surface of bare chitosan (Fig. 9(A)) in the form of cobwebs. The shapes of platelets indicated absence of rupture and disruption to the membranes of platelets. In addition, there were distinguished pseudopodia. As per the description of Bricout *et al.*, all these features are ideal for the activation of platelets.^134^ In contrast, there were a few platelets attached to the surface of CCDF specimen (Fig. 9(B)). The rate of activation of platelets was slower and they covered a small surface area on the specimen. These results were consistent with Nguyen *et al.* which used different materials to reduce the activation of platelets on the surface of specimens.^135^ The CCDF specimens have hydroxyl bonds and acholic functional groups on the surface which prevented platelet adhesion and did not initiate the thrombus formation. Through this study, it was proved that CCDF specimen had potential ability to inhibit the platelet adhesion to the surface of specimens which could be used in different medical applications.

Thrombosis is one of the major reasons for implant failure in the human body. Major precursors of thrombus formation are blood coagulation and platelet adhesion at the target site and if the rate of platelet adhesion is controlled, the coagulation and thrombus formation may be tailored according to the requirements. This study has shown that CCDF specimen has strong anticoagulation properties which reduces the rate of platelet adhesion, and it may be speculated that it would reduce the rate of thrombosis at target site.

Analysis of adhesion of blood cells to the implant surfaces is commonly tested to observe if the surface of implant can resist the formation of thrombus. As shown by Krajewski *et al.*,^136^ both albumin and heparin coatings on cardiac implants reduced blood cell adhesion compared to bare surfaces. Similar results were demonstrated in our study as the drug incorporated in matrix (CCDF) significantly reduces cell attachment, likely due to the anticoagulant drug incorporated into it. Tang *et al.*^137^ reported that cardiac implants tend to adhere more platelets on their surface if these are not coated with any polymeric or drug substance which leads to early thrombus formation at site. In another study, Busch *et al.*^138^ studied various cardiac implants which had coatings of different polymers including PLLA, P(3HB), P(4HB), and a blend of PLLA and P(4HB) and compared their results with each other. The results showed that while PLLA and P(4HB) individually induced thrombosis, their combined formulation exhibited an antiplatelet adhesion effect. This aligns with the results of our observation as combining an anticoagulant drug with a polymer reduces platelet adhesion, ultimately mitigating thrombus formation.

## Conclusions

In conclusion, in this study, plant based novel drug loaded polymeric coatings were explored which may decrease the rate of thrombosis at biomedical device implantation site by blocking the coagulation cascade. The morphological and compositional analysis demonstrated the presence of drug on the surface as well as embedded inside the matrix. The drug particles present on the implant surface were responsible for the initial burst release; however, tightly bound drug particles inside the matrix were released in a slow and sustained manner due to the degradation of matrix. The FTIR analysis demonstrated a strong affinity between drug molecules and chitosan matrix which was responsible for the slower release of drug. These films were found to have good hemocompatibility, anticoagulation and antioxidant properties. The presence of constitutes responsible for anticoagulation were confirmed through GC-MS analysis, and interaction between anticoagulation constitutes and proteins of coagulation cascade were analyzed through molecular docking which indicated blockage of cascade at multiple sites. Considering the results of hemocompatibility and anticoagulation, CCDF was proposed as the suitable candidate for biomedical implant coatings.

## Limitations and future direction

This study was focused on thrombus-related experiments using fresh blood which perfectly fulfilled the established research objectives and project scope. However, there was a lack of investigation of the interaction of these thin films with human cells within a human blood environment due to a lack of specific cell lines in our facility. Future studies will be focused on acquiring these cell lines and doing more experiments to evaluate biocompatibility of these drug-polymer composites.

While GC-MS analysis was able to identify the anticoagulant constituents within the prospective drug, quantification of these anticoagulants was not possible due to lack of specific instrumentation. Thus, future research will be focused on quantification of these constitutes using GC-MS and HPLC. Lastly, this research served as a preliminary investigation which used thin films for initial assessments. In future, more studies will be done to fully evaluate the suitability of the material for real-world biomedical implant applications.

## Ethical statement

All the procedures involving human blood were performed in accordance with the Ethical Guidelines for Collection, Usage, Storage, and Export of Human Biological Materials (HBM) provided by National Bioethics Committee, Pakistan. The institutional review board of Department of Biomedical Engineering and Sciences, School of Mechanical and Manufacturing Engineering, NUST approved the study.

## Author contributions

SH conceptualized the study, designed the methodology and prepared the manuscript. NB was responsible for the data curation and TJK helped in investigation. MNA supervised the study and provided the resources. BG and KUS helped in formal analysis, writing and reviewing the manuscript.

## Conflicts of interest

There are no conflicts to declare.

## Supplementary Material

RA-014-D4RA00796D-s001
