# Supplementary material for: Bioinspired chitosan based functionalization of biomedical implant surfaces for enhanced hemocompatibility, antioxidation and anticoagulation potential: an in silico and in vitro study
Source: RSC Adv. 2024 Jul 1;14(29):20691–713. doi: 10.1039/d4ra00796d (PMC11215499; doi:10.1039/d4ra00796d)
Supplement: RA-014-D4RA00796D-s001 [file RA-014-D4RA00796D-s001.pdf]

Electronic Supplementary Material (ESI) for RSC Advances.
This journal is © The Royal Society of Chemistry 2024

Supplementary Data

Table 1: Details of drug ligands and their bonding with proteins of coagulation cascade along with energy scores

SR #	DRUG	PROTEIN	ENERGY SCORE
**1**	Fenchone	VIIa	- 4.9
**2**	Cinnamaldehyde	Xa	-5.6
**3**	Camphor	Xa	-4.6
**4**	Ferulic acid	Xa	- 4.8
**5**	senkyunolide A	VIIa	- 5.2

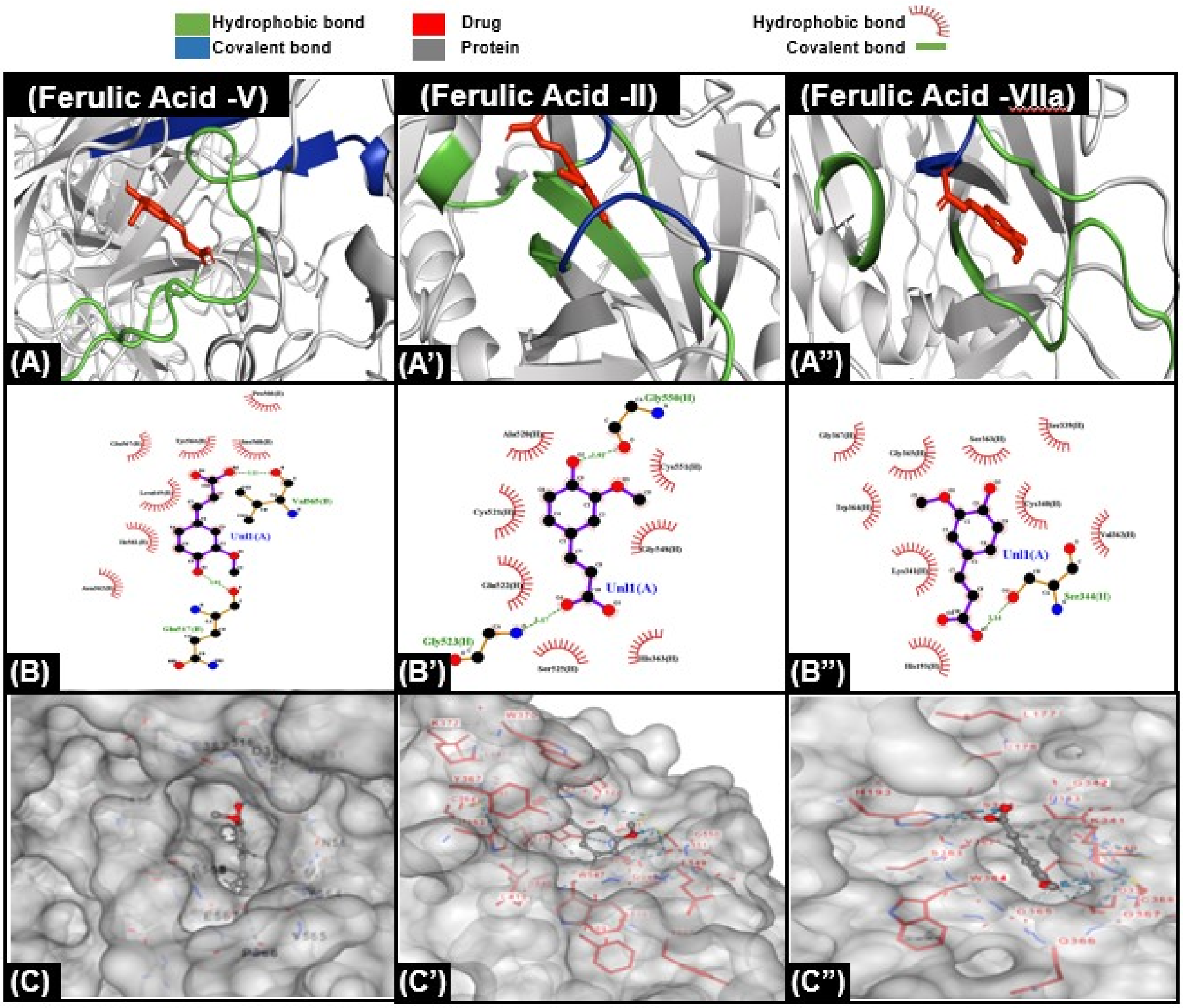

Figure S1: Demonstration of ferulic acid interaction with different proteins of coagulation cascade. These interactions were weaker as compared to its interaction and bonding with factor II.

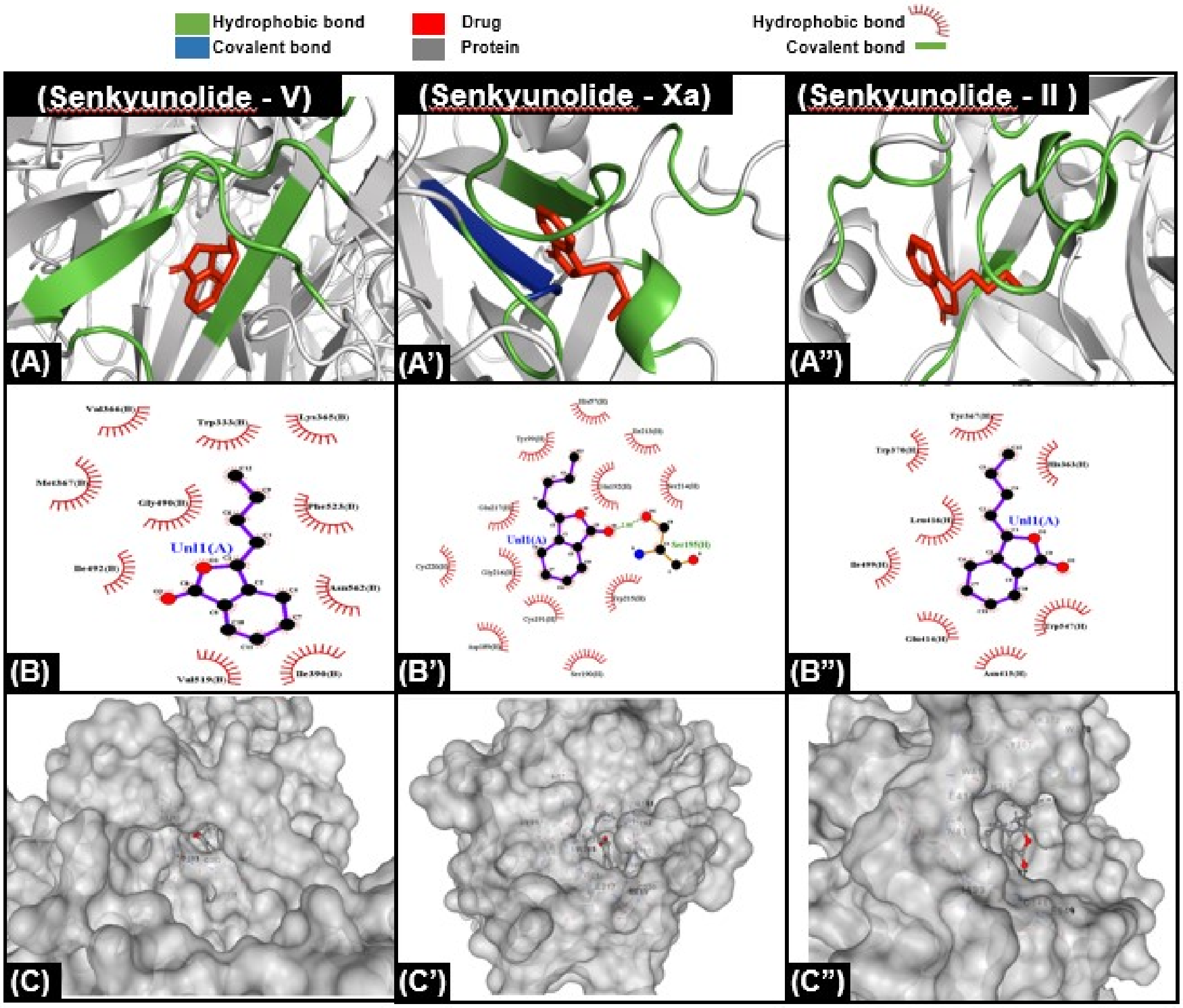

Figure S2: Demonstration of Senkyunolide interaction with different proteins of coagulation cascade i.e., V, Xa and II. These interactions were weaker as compared to its interaction and bonding with factor VIIa due to lesser number of hydrophobic and covalent bonding.

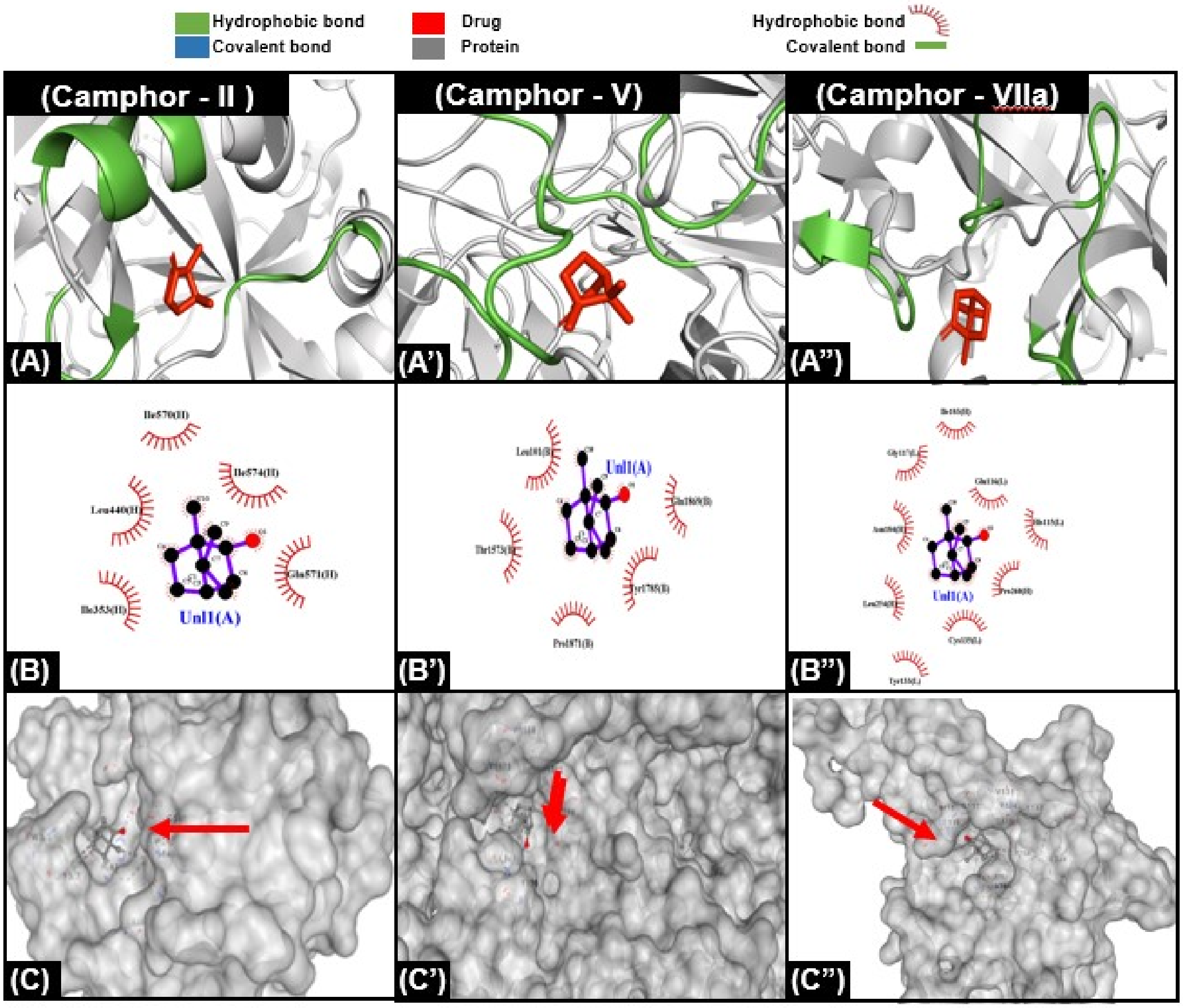

Figure S3: Demonstration of camphor interaction with different proteins of coagulation cascade.

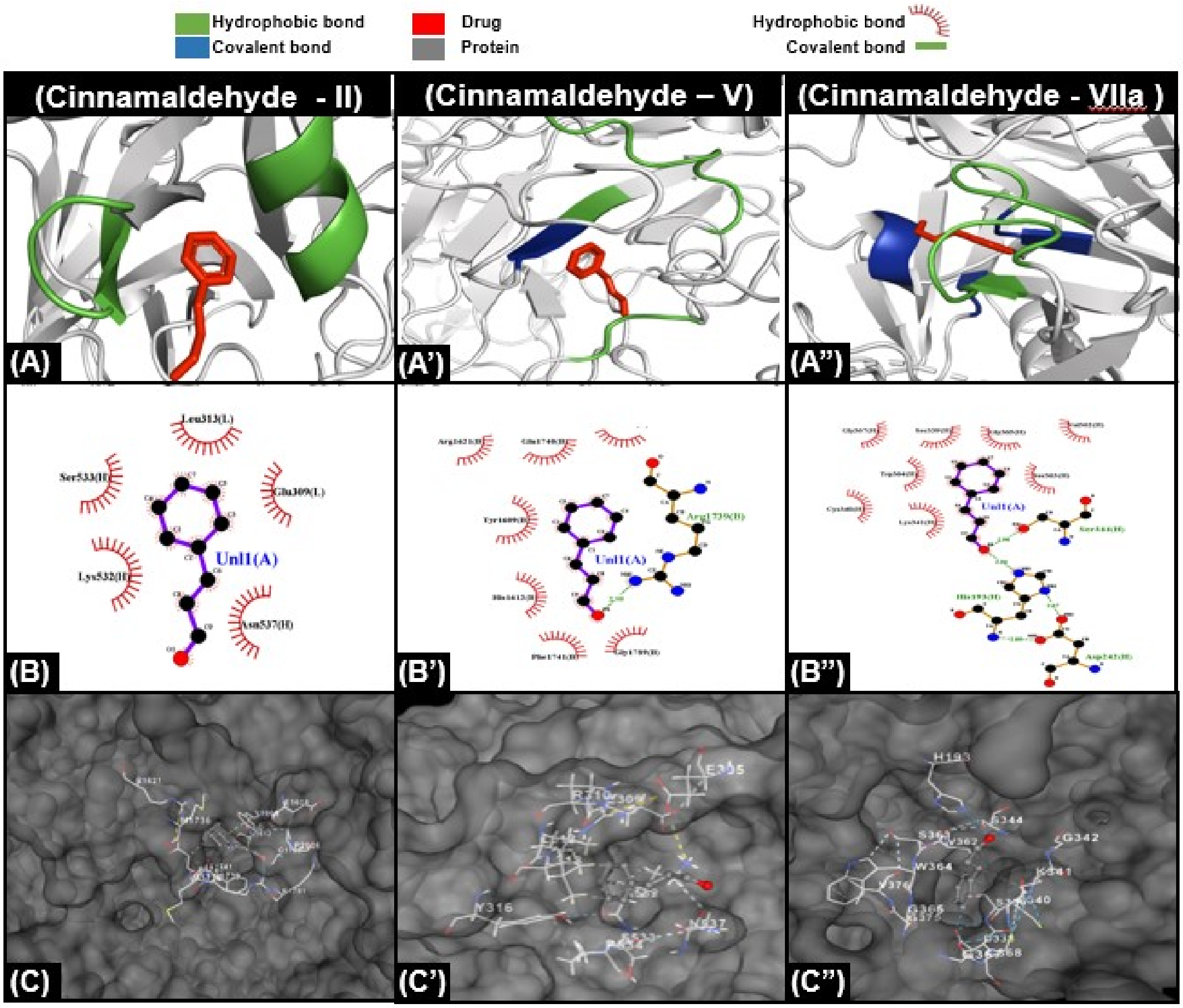

Figure S4: Demonstration of cinnamaldehyde interaction with different proteins of coagulation cascade.

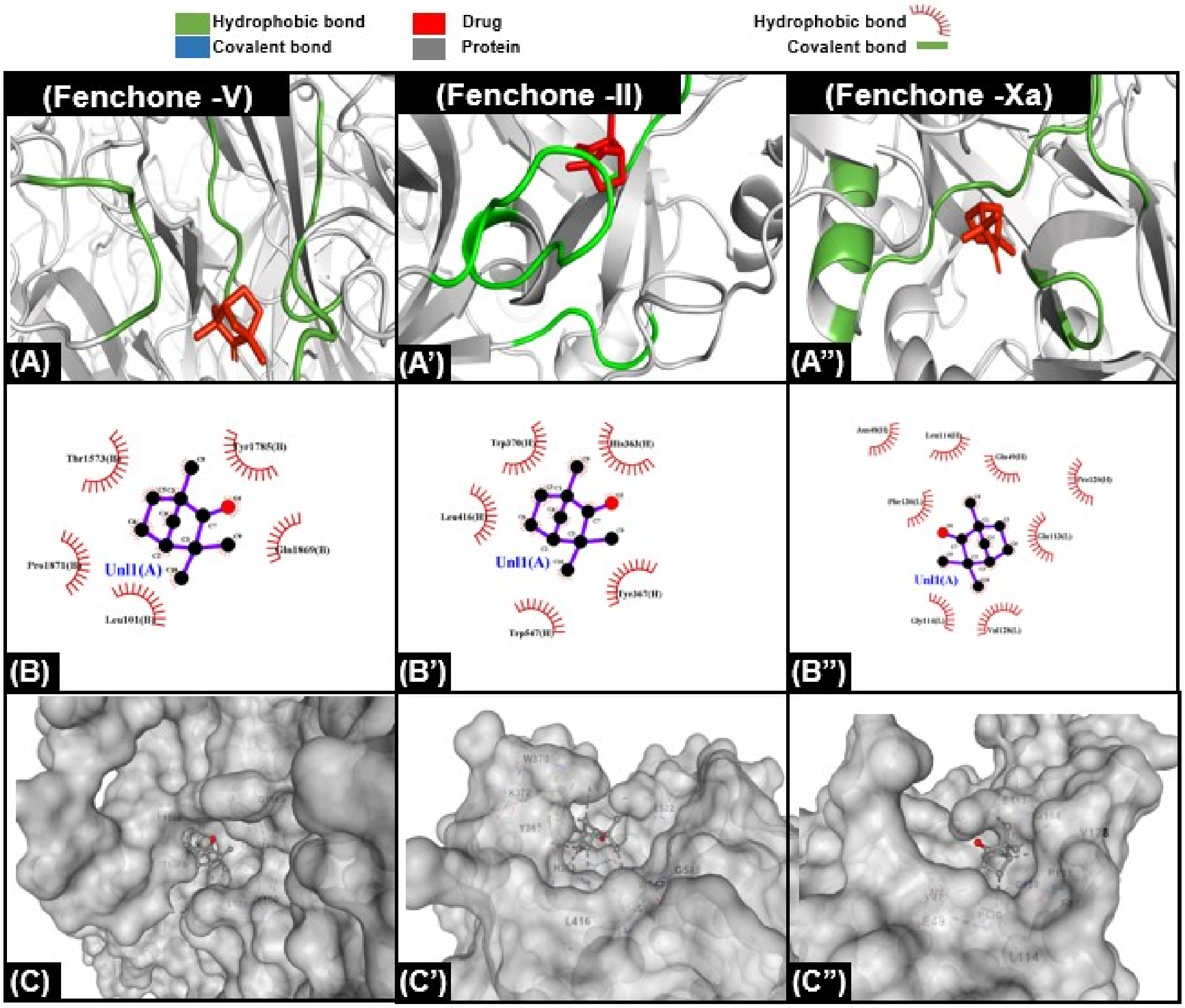

Figure S5: Demonstration of fenchone interaction with different proteins of coagulation cascade.

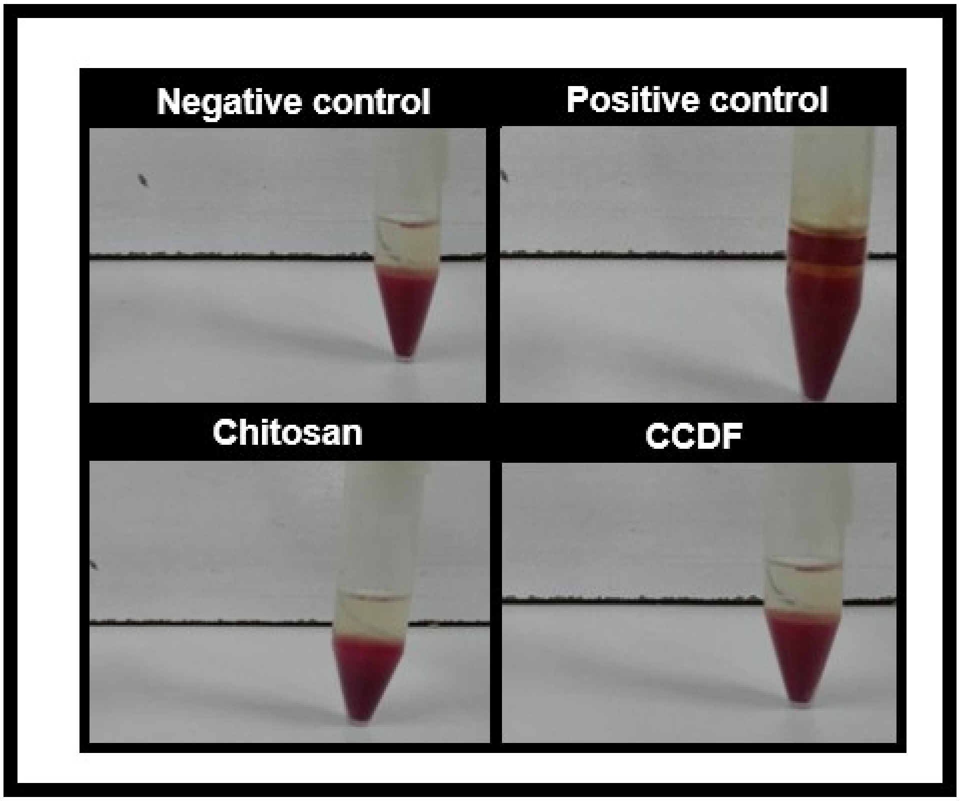

Figure S6: Demonstration of hemolysis test for specimens
